# Electroconductive Bionanocomposites from Black Soldier Fly Proteins for Green Flexible Electronics

**DOI:** 10.1021/acssuschemeng.4c08242

**Published:** 2025-02-03

**Authors:** Edoardo Testa, Vincenzina Barbera, Elisa Fasoli, Ulrich Giese, Maria Rosaria Belviso, Pasqua Rossini, Daniele Bruno, Gianluca Tettamanti, Marco Orlando, Gianluca Molla, Morena Casartelli, Maurizio Galimberti

**Affiliations:** † Department of Chemistry, Materials and Chemical Engineering “G. Natta”, Politecnico di Milano, Via Mancinelli 7, 20131 Milano, Italy; ‡ Deutsches Institut für Kautschuktechnologie e. V., Eupener Straße 33, 30519 Hannover, Germany; § Plasmapps Srl, Via VVF Caduti in Servizio 14, Zona artigianale Modugno, 70126 Bari, Italy; ∥ Department of Biotechnology and Life Sciences, Università degli Studi dell’Insubria, Via J. H. Dunant 3, 21100 Varese, Italy; ⊥ Interuniversity Center for Studies on Bioinspired Agro-environmental Technology (BAT Center), Università di Napoli Federico II, Piazza Carlo di Borbone 1, 80055 Portici, Italy; # Department of Biosciences, Università degli Studi di Milano, Via Celoria 26, 20133 Milano, Italy

**Keywords:** proteins, nanocomposite, black soldier fly, organic waste, flexible electronics

## Abstract

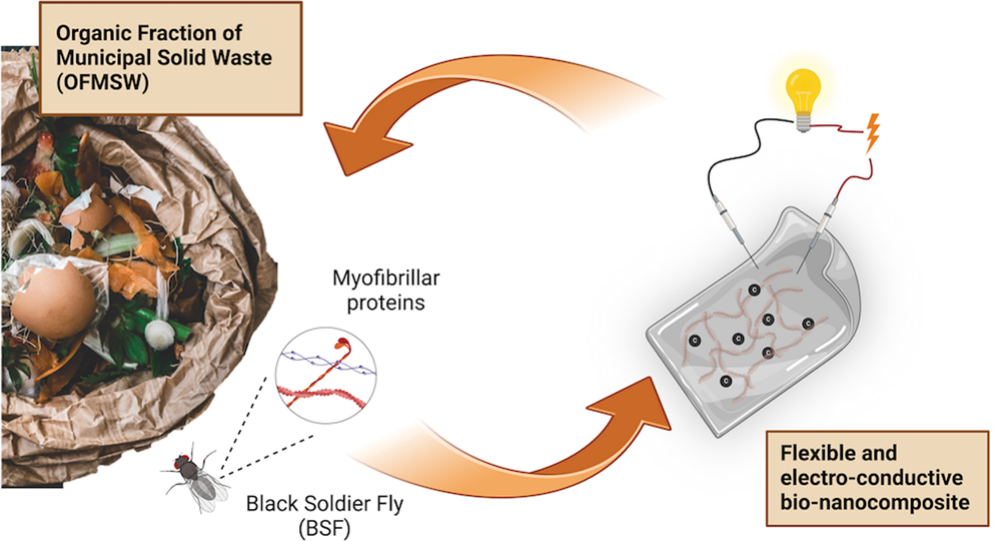

Printed and flexible electronics hold the potential to revolutionize the world of electronic devices. A primary focus today is their circularity, which can be achieved by using biobased materials. In this study, electrically conductive bionanocomposite materials suitable for flexible electronics were fabricated using proteins from the black soldier fly (BSF, *Hermetia illucens*). The valorization of BSF biomacromolecules is currently being pursued in the framework of emerging circular economy models for the bioconversion of the Organic Fraction of Municipal Solid Waste (OFMSW), where BSF has been demonstrated to act as an extremely efficient bioconverter to provide lipids, chitin, and proteins. Here, the BSF protein extracts were characterized by proteomic techniques, revealing a pool of myofibrillar proteins able to interact through intermolecular β-sheet interactions. Flexible and electroconductive bionanocomposite materials were next formulated by combining BSF proteins with a conductive carbon black (CCB), either in its pristine form or functionalized with 2-(2,5-dimethyl-1*H*-pyrrol-1-yl)-1,3-propanediol (serinol pyrrole, SP), using water as the only solvent and incorporating glycerol and carboxymethylcellulose (CMC) as additional green ingredients. A sustainable, low-pressure cold plasma (LPCP) technology was ultimately proposed to achieve high film surface hydrophobicity. Characterized by effective biodegradability, strain-sensing properties, high electrical conductivity (up to 0.9 × 10^–2^ S/cm at a filler content of 8% v/v (15% w/w)), and high surface hydrophobicity, the bionanocomposites presented here may be well suited for disposable flexible electronics, as in wearable devices, electrostatic discharge fabrics, or packaging, hence offering new routes toward OFMSW valorization and the development of green flexible electronics.

## Abstract 2

Research on sustainable bionanocomposite films from insect proteins for the full valorization of the organic fraction of municipal solid waste.

## Introduction

The rapid development of electronics has led to an escalating problem of electronic waste, commonly known as e-waste, whose global production is expected to reach 74.7 million tons by 2030.^1^ This alarming increase is fueled by the increasing consumption and short life span of electronic equipment and encouraged by the use of nondegradable substrates in electronic components, contributing to their accumulation in the environment. For instance, in emerging printed and flexible electronics, newest technologies have already achieved considerable success in producing electrical circuits,^2^ transistors,^3^ solar cells,^4^ and light-emitting diodes (LEDs).^5^ Yet, the substrates used in similar technologies are often made from nonbiodegradable and oil-based polymeric films like polyethylene terephthalate (PET),^6^ polyimide (PI),^7^ polydimethylsiloxane (PDMS),^8^ as well as textiles.^9^

In this regard, green flexible electronics (GFE) are emerging as a new and disruptive class of devices.^10−13^ By leveraging natural polymers such as starch,^12^ paper,^13^ wood,^2^ keratins,^14^ zein,^15^ soy,^16^ whey proteins,^17^ and other biomasses, GFE have so far combined the properties of biodegradability, renewability, and eco-friendliness with the versatility of flexible electronics. These advantages make biobased and biodegradable polymers ideal for applications such as healthcare surveillance,^18−23^ environmental monitoring,^17,24^ smart packaging,^25^ and flexible devices for energy harvesting/storage.^26,27^ Nowadays, it is essential that these biopolymers do not compete with the food chain, and their supply should not strictly depend on animal and plant farming requiring substantial land and water resources, whose preservation is essential for achieving a socioeconomically and environmentally sustainable future.^28,29^ To this end, further efforts are needed to identify more sustainable biomass feedstocks. Furthermore, tackling the low water stability of biopolymers as proteins and polysaccharides is vital to enabling their broader applicability.

In this work, highly hydrophobic nanocomposites suitable for flexible electronics applications were developed by using proteins from the Black Soldier Fly insect (BSF, *Hermetia illucens*). Thanks to the plasticity of its midgut, BSF can effectively bioconvert a multitude of organic substrates and provide useful biomacromolecules, such as lipids, chitin, and proteins.^30,31^ It has recently been demonstrated that BSF can effectively bioconvert the Organic Fraction of Municipal Solid Waste (OFMSW), a substantial biomass resource whose global generation currently accounts for 1.3 billion tons/year.^32^ The efficacy of this technology has been demonstrated and compared with traditional OFMSW treatments, such as composting or anaerobic digestion.^33−38^ BSF-based technologies lead to higher organic mass reduction (up to 80 vs 50% w/w for composting^37^ and 20% for anaerobic digestion^37^) and to lower land occupation (50 vs 185–530 m^2^/ton/day for composting or anaerobic digestion), besides the advantage of processing mixed organic waste, avoiding the use of much freshwater.

In a recent work, some of the authors reported the potential of BSF as bioconverter of real OFMSW substrates.^39^ The technical, environmental, and socioeconomic relevance of this technology was proven as a circular bioeconomy model, yielding valuable bioproducts such as proteins, lipids, and chitin. In detail, 1 kg of s-OFMSW yielded a total of 48.7 g of BSF pupae, containing 6.3 g of proteins, together with 20.4 g of lipids and 2.0 g of chitin.^39^ In the same work, it was shown that substantial improvements of the bioplastic film properties were achieved with respect to the current literature.^40−42^ However, the low water stability as well as the lack of functionality of proposed materials still hamper their widespread applicability.

In this work, BSF pupae proteins were utilized as the main constituent of a nanocomposite matrix, in combination with a functionalized Conductive Carbon Black/serinol pyrrole (CCB/SP), carboxymethylcellulose (CMC), and glycerol as a plasticizer. Films were prepared via casting, and the filler–matrix dispersion, degradability, and electrical properties were thoroughly investigated. A sustainable low-pressure cold plasma (LPCP) treatment was further proposed to provide materials with high surface hydrophobicity, thereby enhancing their water stability and hence their applicability.

The approach shown in this study not only creates valuable opportunities to upcycle the OFMSW into high-added-value materials but also can foster the advancement of more sustainable and green electronics.

## Results and Discussion

### Characterization of the BSF Protein Extract

**Figure 1:**
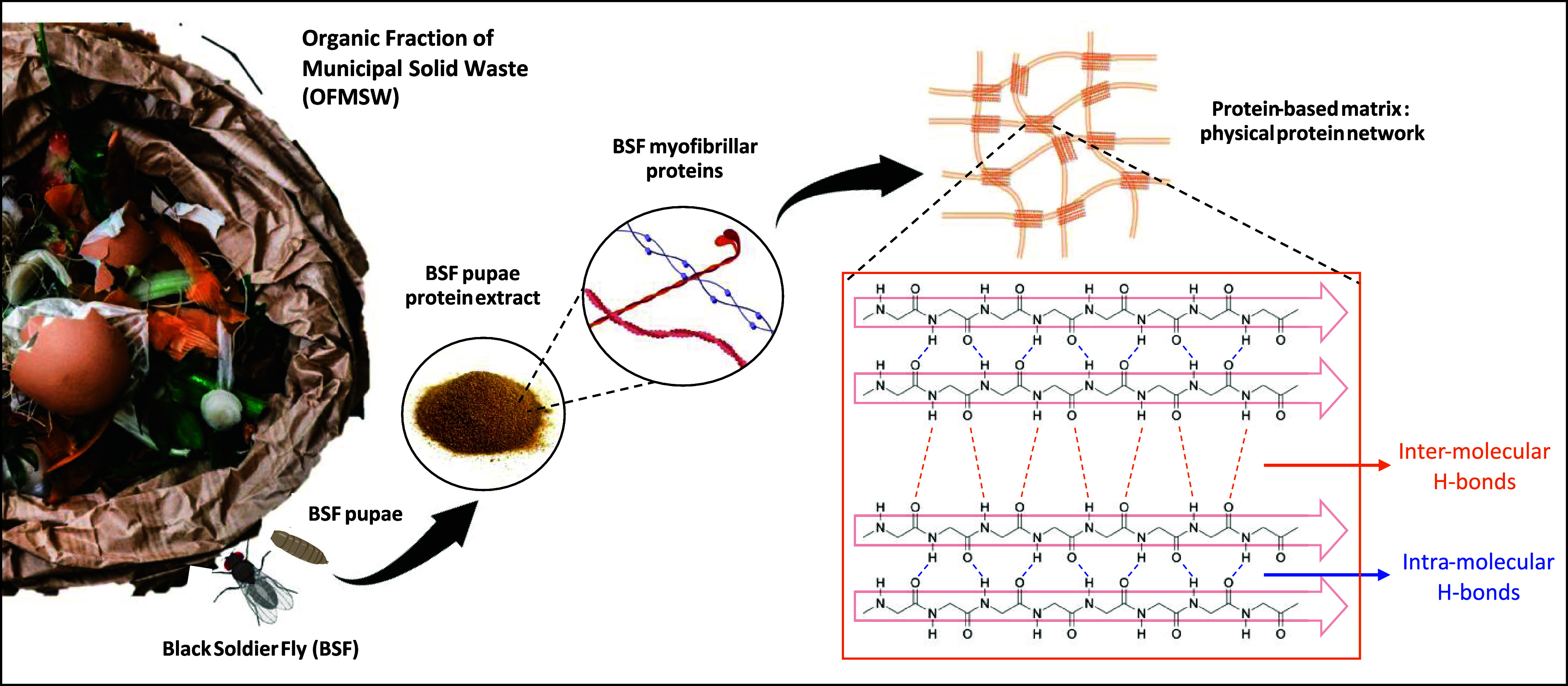
Schematized pathway going from OFMSW to BSF protein-based networks. OFMSW is used as a rearing substrate for BSF larvae. Protein extracts are obtained from the pupal stage of the insect through a process which can be synthesized in three main steps, as described in a recent publication:^39^ freezing and grinding of the insect, defatting of the resulting powder, and protein recovery through isoelectric precipitation. Obtained extracts contain a pool of proteins with different MW, mainly myofibrillar proteins. These proteins can interact through noncovalent interactions between intermolecular β-sheets (H-bonds), thus leading to the formation of a network [Copyright: Galimberti, M. 2024, BioRender.com].

The protein extracts were in the form of a light brown powder (Figure 1), with a soluble protein content of approximately 80–85% of its total weight, as determined by bicinchoninic acid assay (BCA) assays. From a scale-up perspective, single protein purification was not carried out to avoid excessive product costs. Consequently, the utilized BSF protein extracts were characterized by a pool of different proteins. Their molecular weights (MW), according to sodium dodecyl sulfate-polyacrylamide gel electrophoresis (SDS-PAGE) analysis, ranged between 10 and 250 kDa (Figure S1). Specifically, intense bands in the gels were detected around 75, 50, 30, and 20 kDa as well as in the region above 180 kDa. nLC-MS/MS analyses, focusing on bands up to 75 kDa, disclosed a predominant presence of structural proteins, such as myofibrillar proteins, specifically belonging to myosin, tropomyosin, actin, and troponin families, along with a few enzymes (Tables S1 and S2).

Interestingly, BSF proteins were found to undergo ordered aggregation in alkaline water solutions (pH 12) by forming supramolecular β-sheet interactions, as detected by ThioflavinT (ThT) fluorescence analysis (Figure S2). Commonly used to evaluate the formation of amyloid-like supramolecular aggregates,^43,44^ the ThT assay was used as a tool to monitor the evolution of this type of interactions between BSF proteins. The development of such structures is highly significant in the field of protein-based materials since they can impart mechanical robustness and water stability to the final material.^17,45−47^ Here, the development of the characteristic ThT fluorescence emission peak at λ_em_ = 490 nm was observed for suspensions of BSF protein resuspended in a 0.1 M NaOH solution (Figure S2A), confirming their propensity to self-assemble via intermolecular β-sheets under the film-forming conditions. The fluorescence intensity increased over time, reaching a plateau after about 8 h. Notably, this increase was not observed for solutions at pH 2 (i.e., a common condition for protein fibrillation)^48^ (Figure S2B). These results are in accordance with our previous study, where a significant component associated with intermolecular molecular β-sheets interactions was detected by ATR-FTIR (1624–1615 cm^–1^ signal) in Bioplastic films made from a combination of BSF proteins and glycerol.^39^

Together, these results confirm that BSF pupa protein extracts are complex mixtures of proteins with different native functions and conformations. The dissolution of this protein mixture in alkaline environments is feasible and can promote the formation of supramolecular physical networks, which can be exploited as a matrix for composite materials.

### Preparation and Characterization of the Adduct CCB/SP

**Figure 2:**
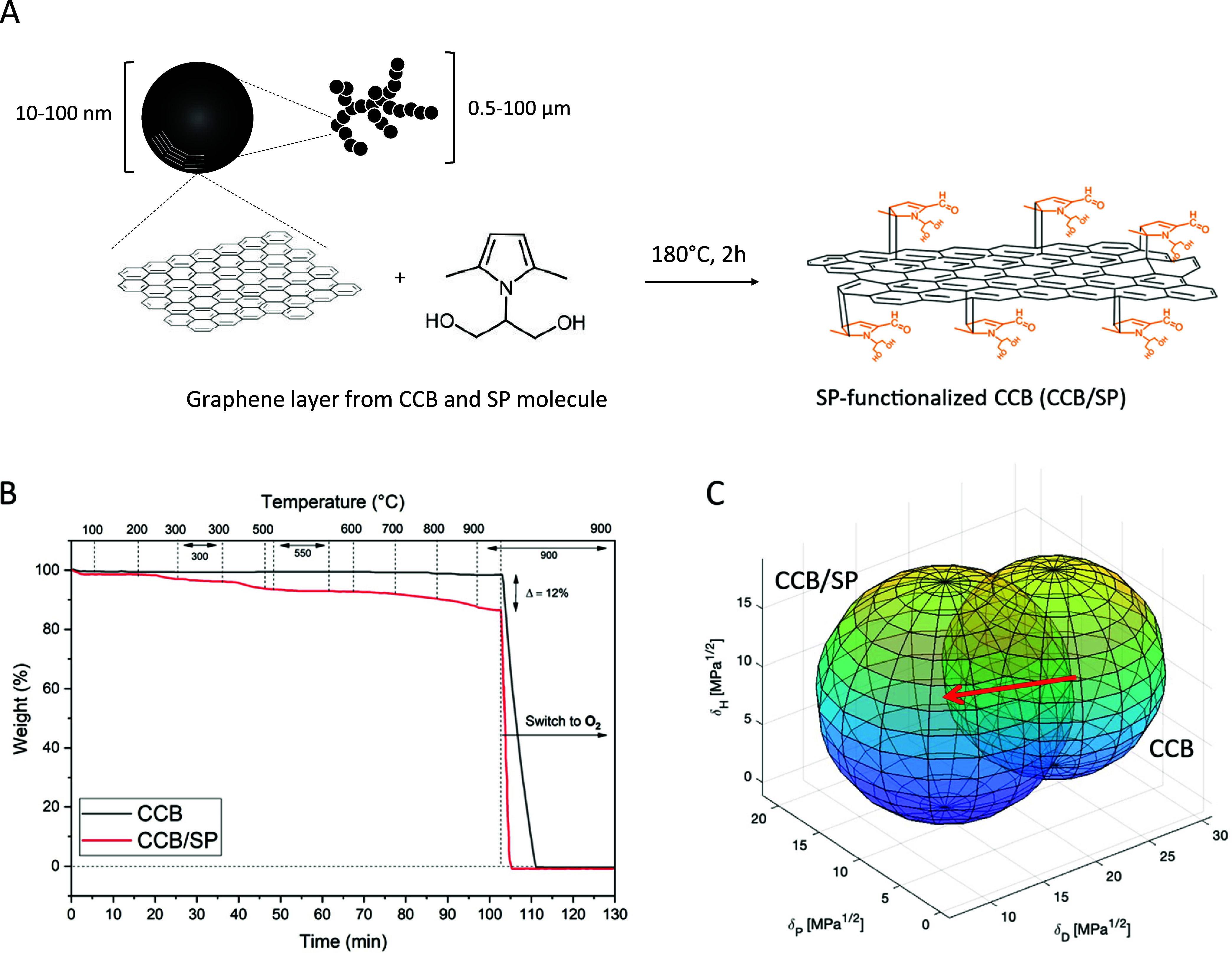
Preparation and characterization of the adduct CCB/SP. (A) Schematic representation of CCB/SP adduct formation and chemical species involved in the functionalization process. (B) Thermogravimetric curves of CCB (black) and CCB/SP (red curve). (C) Graphical representation of the Hansen spheres for CCB and CCB/SP. The polarity increase after functionalization is clearly highlighted by a shift (red arrow) toward higher δ_P_ and lower δ_D_ (see Table 2 for numeric values).

Owing to their excellent electrical and mechanical properties, lightweight, chemical stability, and relatively low price, sp^2^ carbon allotropes (i.e., graphene, graphites, carbon nanotubes, or carbon blacks) are usually the preferred choice of conductive filler in composite materials requiring moderate to high electrical conductivity (σ).^10,12,49,50^ So far, these nanomaterials have been integrated either with oil-based or biobased polymer matrices to give flexible and electroconductive composites.^13,14,17,51−60^ However, their full potential as conductive elements in films or inks for printed electronics may be quenched due to their low polarity, ultimately leading to particle aggregation. Here, the polarity of CCB was increased by a functionalization process with serinol pyrrole (SP), providing the adduct CCB/SP (Figure 2A). This process is easy and sustainable and avoids the use of catalysts or toxic solvents (Figure S3 and Text S3). Mechanisms underlying this functionalization step have been comprehensively elucidated in prior investigations by some of the authors^61^ (Text S4).

The assessment of CCB functionalization with SP was conducted through thermogravimetric analysis (TGA). Thermograms of CCB and the CCB/SP adduct are reported in Figure 2B, and their interpretation and relative weight losses are reported in Text S6 and Table S3, respectively. After a primary weight loss below 150 °C, attributed to adsorbed water molecules, the analysis revealed a weight loss of approximately 12.1% in the temperature range of 150–900 °C for the CCB/SP adduct. In contrast, pristine CCB exhibited a weight loss of only 1.1% in the same temperature range. A degree of functionalization (DoF, %) of 11% on the total weight of the adduct was thus determined for CCB/SP. The calculated functionalization yield (FY) associated with the grafting reaction was 58%.

The crystalline structure of the obtained CCB/SP was investigated by XRD analysis. Except for a slight widening and lowering of the CCB/SP (002) reflection peak, diffractograms presented in Figure S4 indicated minimal changes in both *in-plane* (100) and *out-of-plane* (002) orders when comparing the two species. As proposed in prior studies,^61−64^ this outcome is likely associated with edge functionalization of graphene layers, where defects are more present and act as highly chemically reactive sites. Consequently, the diffractogram of the CCB/SP adduct, and by extension, the main crystalline structure of the carbon substrate, appeared to be unaffected. Notably, this was a sought-after result since the preservation of the crystalline structure of CCB theoretically enables the preservation of its original conductivity.

To ascertain whether SP functionalization resulted in an affinity shift of the adduct toward higher polarity, thus promoting stronger filler–matrix interactions, the Hansen surface parameters of CCB and CCB/SP were computed and evaluated. Hansen parameters are commonly used to predict the solubility of materials in solvents (Hansen Solubility Parameters, HSP), particularly for polymers. For insoluble materials, as in the case of CCB, Hansen *surface* parameters can be obtained from precipitation stability tests of suspensions. In this work, suspensions of CCB and CCB/SP were prepared in solvents with varying HSP values, and their precipitation stability was visually examined (Figure S5) and recorded (Table 1). Hansen surface parameters and associated spheres were then calculated and elaborated as described in the Experimental Section (Figures 2C, S6 and Table 2). The obtained results clearly evidenced distinct Hansen surface parameters for the two fillers. A significant shift of the sphere center toward higher δ_P_ and lower δ_D_ occurred for the CCB/SP adduct, suggesting an improved affinity for the CCB/SP surface toward polar ingredients (e.g., proteins). Conversely, δ_H_ appeared to be relatively unaffected by the functionalization process.

**Table 1:** Dispersibility of CCB and CCB/SP in Solvents with Different HSP^a^

				dispersed (yes = 1, no = 0)	
solvent	δ_D_ (MPa^1/2^)	δ_P_ (MPa^1/2^)	δ_H_ (MPa^1/2^)	CCB	CCB/SP
water	18.1	12.9	15.5	1	1
acetone	15.5	10.4	7	0	1
ethyl acetate	15.8	5.3	7.2	1	1
hexane	14.9	0	0	0	0
chloroform	17.8	3.1	5.7	1	1
dichloromethane (DCM)	17	7.3	7.1	1	1
methanol	14.8	12.3	22.3	0	0
1-butanol	16	5.7	15.8	1	0
tetrahydrofuran (THF)	16.8	5.7	8	1	1
2-propanol	15.8	6.1	16.4	0	1

a Data from this table were used to compute the Hansen surface parameters and associated spheres.

**Table 2:** Hansen Surface Parameters Associated with Spheres in Figure 2B for CCB and the CCB/SP Adduct

	δ_D_ (MPa^1/2^)	δ_P_ (MPa^1/2^)	δ_H_ (MPa^1/2^)	radius	δ_T_ (MPa^1/2^)
CCB	22.5	7.4	10.9	8.4	26.0
CCB/SP	15.9	12.2	9.6	9.8	21.8

### Preparation of BSF Protein-Based Bionanocomposite Films

**Figure 3:**
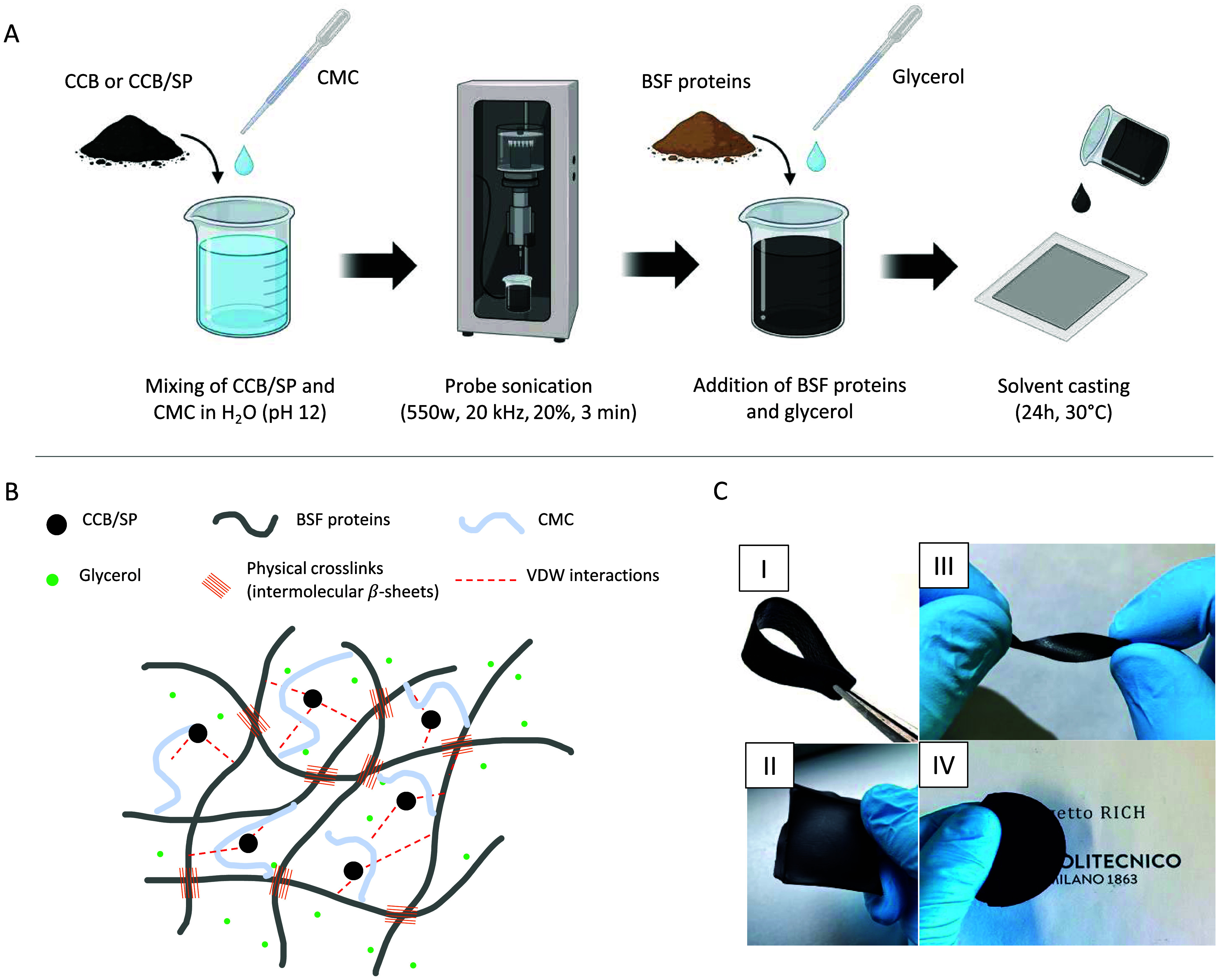
Preparation of BSF protein-based bionanocomposite films. (A) Schematization of the lab-scale procedure for the preparation of flexible BSF protein-based electroconductive nanocomposites [Copyright: Galimberti, M. 2025, BioRender.com]. (B) Schematic representation of the chemical species and interactions involved in the formation of the physical network of the nanocomposite. (C) Visual appearance of the prepared bionanocomposite: the material is free-standing and highly flexible.

The BSF protein-based bionanocomposites were prepared through a straightforward process detailed in the Experimental Section and schematized in Figure 3A, with formulations as in Table S4. Briefly, the CCB/SP adduct (or CCB) and CMC were first dispersed in a pH 12 Milli-Q water solution by tip sonication to obtain a homogeneous mixture. Then, BSF protein extracts were added along with glycerol. The mixture was next stirred for 10 min at room temperature (RT) (20 ± 2 °C) and cast onto PDMS molds to allow solvent evaporation and film formation. The process yielded free-standing films formed by the creation of a physical network of supramolecular β-sheet interactions between BSF proteins, with glycerol acting as an external plasticizer and CMC acting as a stabilizer for CCB (Figure 3B).

BSF proteins constituted the main element of the composites, while glycerol and CMC were added in weight ratios (BSF/glycerol and BSF/CMC) of 100:50 and 100:20, respectively. CCB or CCB/SP was added at contents ranging from 0 to 25 ppm matrix (phm), where the matrix was constituted by BSF proteins and CMC (see Table S4). Further experimental details about the influence of each procedure step and the ingredients used on the final outcome are reported in Text S7.

The obtained bionanocomposite films were black, opaque, and highly flexible (Figure 3C), with thicknesses ranging from 140 to 230 μm.

### Characterization of the Nanocomposites’ Filler–Matrix Interaction

The filler–matrix interaction in the bionanocomposites was investigated through the Kraus plot analysis, derived from films’ water absorption gravimetric data, and transmission electron microscopy (TEM) analysis, carried out directly on micrometric slices of the bionanocomposite’s specimens.

**Figure 4:**
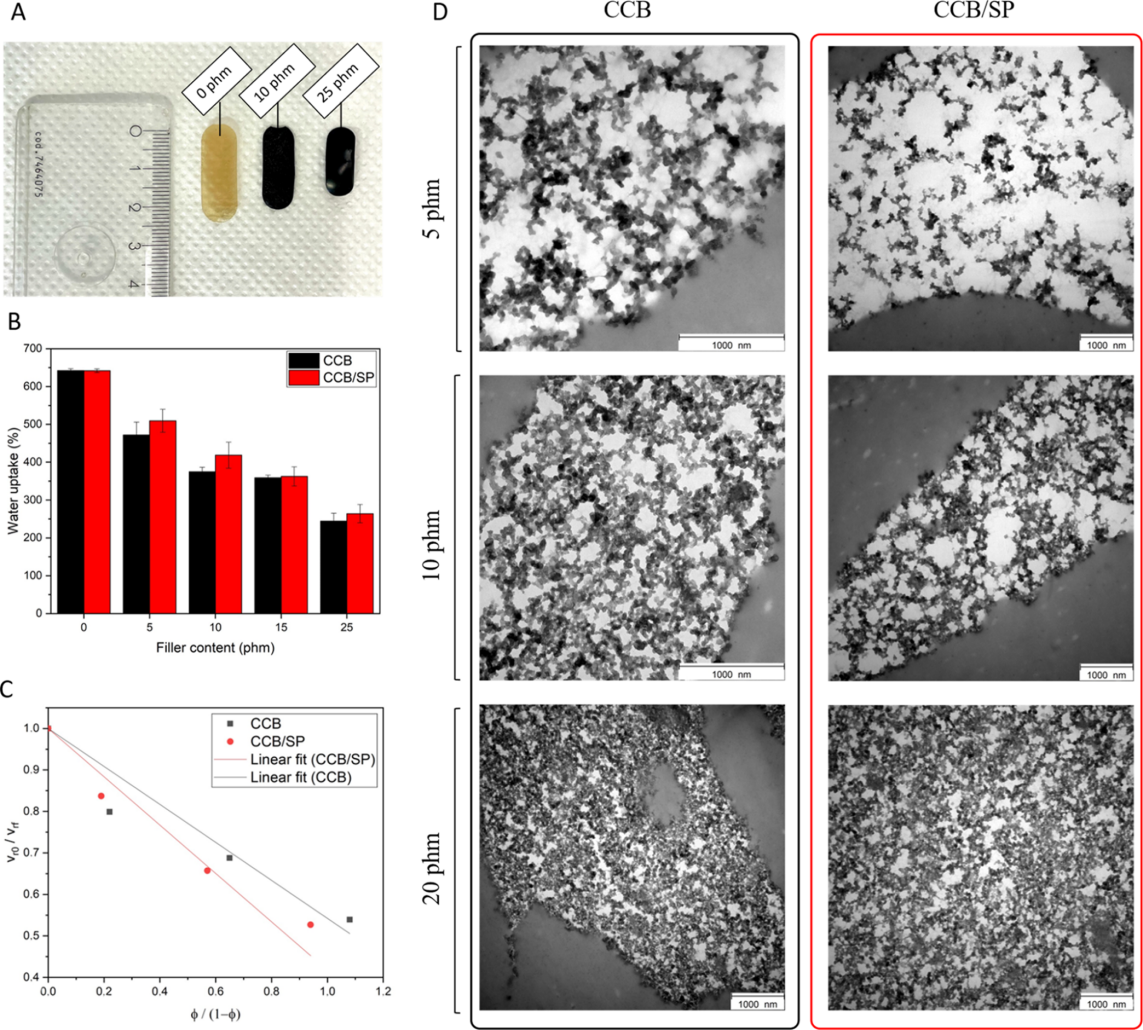
Evaluation of the filler–matrix interaction and dispersion. (A) Bionanocomposite samples after 2 h of immersion in water evidencing different volumetric variations depending on filler content (0, 10, and 25 phm). (B) Water uptake (% w/w) as a function of the filler content for both sets of bionanocomposite films (black columns for CCB and red columns for CCB/SP). (C) Kraus plots for the evaluation of the filler–matrix interaction in CCB and CCB/SP-containing bionanocomposite films. Continuous lines represent the linear fitting of experimental data (black and red dots) according to the Kraus equation (see Text S9, eq 10). (D) TEM micrographs of micrometric slices (obtained by cryo-ultramicrotome) of CCB and CCB/SP-containing BSF protein-based nanocomposites. Samples with different loadings (5, 10, and 20 phm) of both types of nanofillers are displayed. Scale bar: 1000 nm.

Gravimetric data, gathered from swelling tests, showed a moisture content (M.C.%) between 15 and 20% for all of the starting bionanocomposite films. When dried and submerged in water, the films displayed varying water uptake (WU%) depending on the filler content. Specifically, films with higher filler content exhibited lower volumetric variation (Δ*V*%) (from 350 to 150%, ranging from 0 to 25 phm CCB and CCB/SP composites) (Figures 4A and S7) and WU (from 650 to 250%, ranging from 0 to 25 phm CCB and CCB/SP composites) (Figure 4B). A similar trend was observed for film solubility (FS%), with lower solubility values for films with higher filler contents (from 52 to 38%, ranging from 0 to 25 phm composites) (Figure S8 and Table S5). Under the adopted experimental conditions (i.e., water at neutral pH and room temperature), the soluble components of the composites were presumably glycerol and a small fraction of proteins which did not take part in the matrix network, as reported in a previous publication for films composed solely of glycerol and BSF proteins.^39^ In addition to glycerol and proteins, CMC could also have been extracted, to some extent, being CMC water-soluble.

Overall, gravimetric data suggested no significant differences between films containing either CCB or CCB/SP. However, when considering the lower content of CCB in the CCB/SP adduct (Table S4) and the higher polarity of CCB/SP (see Hansen solubility parameters in Table 2), higher WU values would have been expected for CCB/SP-containing composites. This outcome could be attributed to a stronger filler–matrix interaction for composites containing the functionalized filler. To test this hypothesis, experimental data were elaborated through the Kraus plot analysis, which is usually applied to assess the polymer–filler interaction in elastomeric composites^65−67^ and is here applied for the first time (to the best of our knowledge) to protein-based materials. Details are given in Text S9. The linear fitting of experimental gravimetric data according to the model is depicted in Figure 4C, while outputs associated with the analysis are in Table 3. A higher Kraus constant was found for the bionanocomposites containing CCB/SP. Hence, according to the analysis, it is possible to confirm that functionalized CCB (i.e., CCB/SP) imparted a higher degree of polymer–filler interaction.

**Table 3:** Outputs from Kraus Plot Analysis^a^

	*m*	*R*^2^	*C*
CCB	–0.42	0.94	0.83
CCB/SP	–0.54	0.97	0.91

a Slope of the computed linear fitting: *m*; square of the linear regression coefficient: *R*^2^; Kraus constant: *C*.

The filler–matrix dispersion was further investigated by TEM analyses. TEM micrographs shown in Figure 4D are representative of the nanostructure and filler–matrix dispersion of bionanocomposites based on either CCB or CCB/SP. Filler agglomerates cannot be detected in any of the micrographs. Thickening of the network was noticed by increasing the loading of the filler. Differences between the networks constituted by CCB or CCB/SP could be hardly noted; only at low filler loading (5 phm) did the dispersion of the CCB/SP adduct appear slightly better, as suggested by wider spacing between particles, compared to CCB. This evidence suggests a more homogeneous dispersion of the CCB-SP particles in the composites.

### (Bio)degradability of BSF Protein Nanocomposites

**Figure 5:**
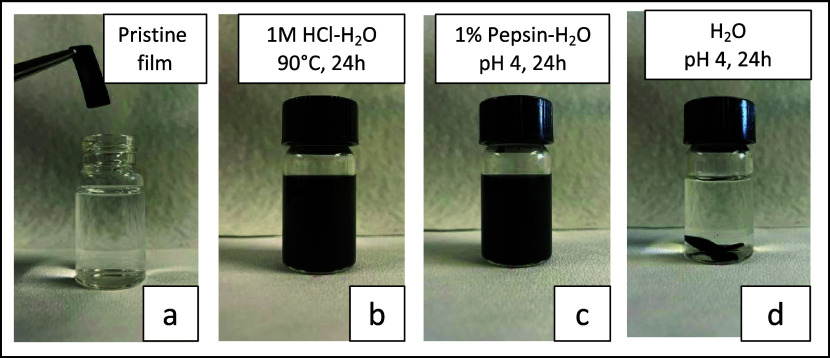
(Bio)degradability test of BSF protein nanocomposites. Visual appearance of the bionanocomposite film containing 25 phm of CBC/SP, before and after the degradation tests: (a) pristine film; (b) after 4 h in a 1 M HCl water solution at 90 °C (c); after 24 h in a 1% pepsin water solution (pH = 4) at 36 °C; (d) after 24 h in a water solution (pH = 4) at 36 °C, without pepsin.

Despite relevant water absorption, primarily due to the high hydrophilicity of the material’s components, and the absence of chemical cross-linking (i.e., only physical cross-links are present, Figure 3B), all films maintained their integrity after at least 1 week of immersion under stirring. This behavior could be attributed to the formation of a pervasive network of intermolecular β-sheets, as commented above, which provides apolar domains that are not easily soluble in water. Degradation of the films was triggered by exposing the samples to either strong acidic or enzymatic water solutions. The results of the experiments are shown in Figure 5. The nanocomposite containing 25 phm of CBC/SP (pristine film in Figure 5a) was degraded within 4 h in a 1 M HCl solution at 90 °C (Figure 5b), or within 24 h by a 1% w/v pepsin solution at 36 °C (Figure 5c). It is worth adding that the control test, carried out in the absence of pepsin under the same experimental conditions, did not lead to any degradation (Figure 5d). After degradation, CCB was successfully recovered by filtration, as demonstrated in Figure S9. The test with pepsin revealed the potential biodegradability of the sample, as previously suggested in the literature.^17^

### Electrical Properties of BSF Protein Bionanocomposites

**Figure 6:**
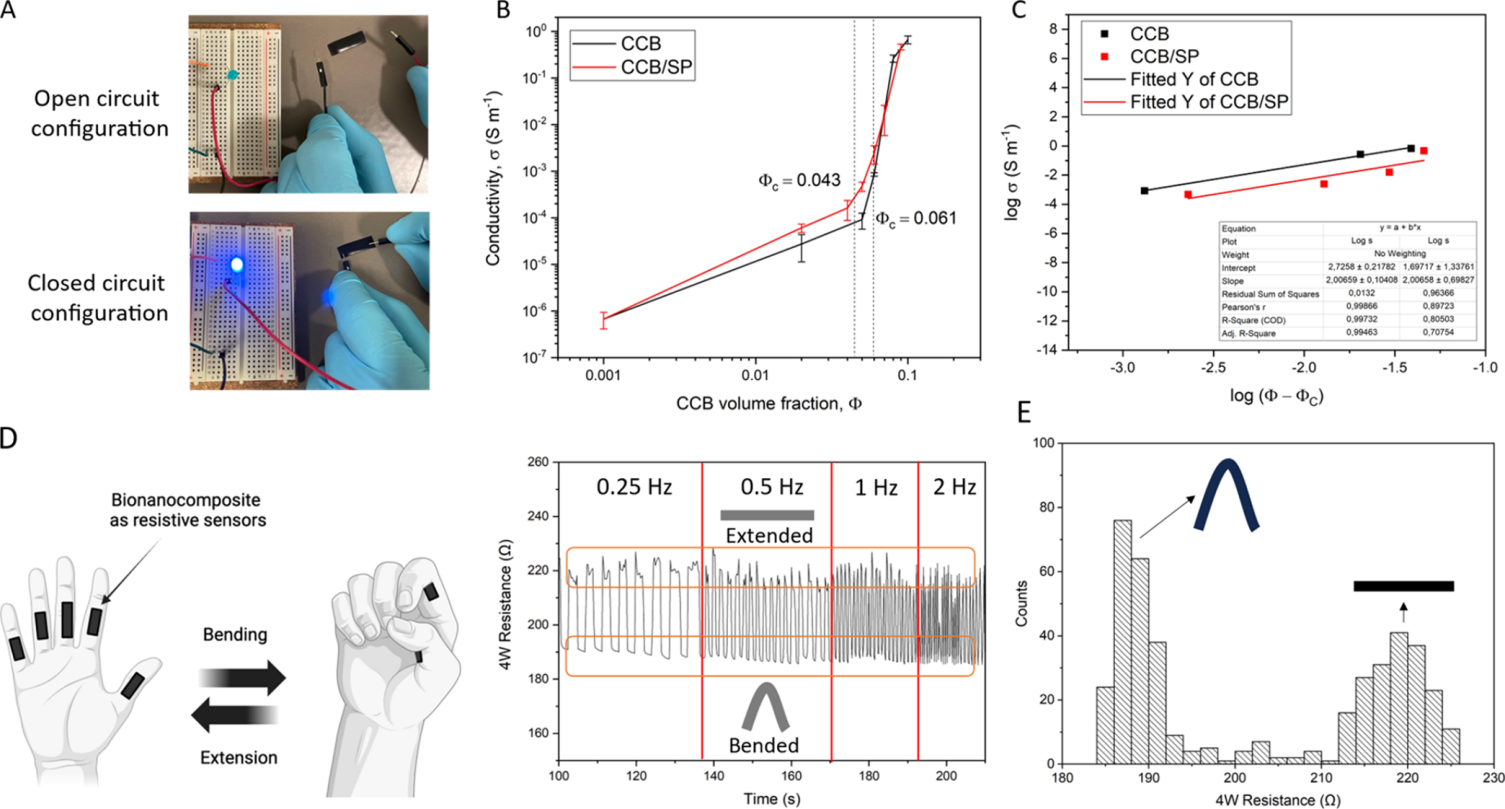
Electrical properties of BSF protein-based nanocomposites. (A) Specimen of the 25 phm CCB/SP bionanocomposite used as a conductive/resistive element in an LED circuit. (B) Electrical conductivity of bionanocomposites as a function of the CCB content (volume fraction, Φ). Electrical percolation thresholds (Φ_C_) are reported for both sets of materials. (C) Linear fitting of electrical conductivity data for CCB and CCB/SP-containing nanocomposites according to percolation theory model. Linear regression coefficients are reported in the inset table. (D) Finger bending mimicking test. The graph on the right reports 4-wire resistance measurements of the 25 phm CBC/SP bionanocomposite film under repeated bending at different bending frequencies. [Copyright: Galimberti, M. 2025, BioRender.com]. (E) Frequency counts of the 4-wire resistance measurements recorded during the cyclic bending test on the composite containing 25 phm CBC/SP.

The bionanocomposite materials prepared in this work displayed appreciably high electrical conductivity, with ranges that well reflect those required by electromagnetic shielding and antistatic applications,^68,69^ as well as those for circuit’s resistive elements in flexible electro-mechanical/chemical sensors.^13,14^ For the sake of demonstration, Figure 6A shows a rectangular specimen taken from the 25 phm CCB/SP bionanocomposite film, used as a resistive element in an LED circuit. Values of electrical conductivity (σ) ranged from 1.50 × 10^–9^ to 0.9 × 10^–2^ S/cm, depending on the filler type and content (Figure 6B).

Notably, the functionalization of CCB with SP appeared to play a beneficial role, promoting a higher electrical conductivity in the medium range of filler contents (5 phm < σ > 20 phm), becoming less evident at high filler contents due to saturation effects.

Basically, conduction in composite materials relies on the mobility of electrons through connected pathways provided by the electroconductive phase (e.g., CCB or CCB/SP). According to *rule of mixture* models, the total electrical conductivity can be approximated as the sum of the conductivities of each component, considering their relative amount.^70^ However, such models pose some limitations as they do not take into account effects related to particle shape and filler–matrix dispersion. These factors are considered for instance in percolation theories.^13,70,71^ Details about this theory and its application in this study are given in Text S11. The same contents of CCB and CCB/SP yielded indeed different conductivity profiles. For both sets of bionanocomposites, these profiles followed with good approximation the percolation model (Figure 6C) described by the following equation
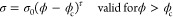
where σ is the electrical conductivity of the composite, σ_0_ is a proportionality constant characteristic of the material, ϕ is the filler volumetric fraction, ϕ_c_ is the percolation threshold, and τ is the universal exponent, with typical values ranging from 1.6 to 2.0 in three-dimensional systems.^13,71^ Given τ = 2, from linear fitting of the experimental data, percolation thresholds of Φ_C_ = 0.061 and Φ_C_ = 0.043 were determined for CCB and CCB/SP-containing bionanocomposites, respectively, in line with the graphical data of Figure 6B.

Such a significant increase in electrical conductivity of a polymer nanocomposite, due to the functionalization of CB, has been also reported in the previous literature.^60^ Indeed, functionalization of CB was shown to improve the CB dispersion in a polypropylene matrix due to the enhanced interfacial interaction. Diarylcarbene derivatives were used, followed by the azocoupling of substituted diazonium salts. The electrical conductivity increased to 1.19 × 10^–4^ S cm^–1^ (at 10 wt % as CB content) from 2.62 × 10^–15^ S cm^–1^ for pristine CB.

It is worth comparing the electrical conductivity of the bionanocomposites of the present paper with that of composites for flexible electronics, reported in the literature, based on sp^2^ carbon allotropes and either biobased or oil-based polymers (Table S6). In all of the biobased composites, graphene-based materials or single-wall carbon nanotubes (SWCNT) were used as the carbon allotrope, whereas CB and graphite were used for the composites with the oil-based polymers. Several composites reported in the literature were based on proteins, such as keratin, soy proteins, and β-lactoglobulin, and contained graphene-based materials. Noteworthy, the electrical conductivity of the BSF-based nanocomposites of this work was found comparable to that of CB-based composites at similar filler loadings.

To further validate the electrical properties of the material, the 25 phm CCB/SP bionanocomposite film was tested as a flexible strain sensor in a proof-of-concept demonstration. Flexible strain sensors have made indeed significant progress, with resistive sensors emerging as a popular choice due to their simplicity, affordability, and strain-detection capabilities via resistance changes caused by deformation.^49^ These sensors work by converting mechanical strain, such as bending or stretching, into proportional changes in resistance, driven by geometric adjustments (e.g., length and cross-sectional area), and/or disconnections in the conductive pathway (loosening of overlapping conductive areas, microcracks, or widening of gaps for tunneling effects).^49^ Upon the release of applied strain, the resistance typically must revert to its initial state, thus, demonstrating reversibility.

Here, dynamic tests mimicking finger bending gestures were carried out on the film to prove its sensitivity to bending deformations (Figure 6D). The film exhibited a significant resistance variation (∼16%) and excellent reversibility over the whole test, with normal distributions of recorded data around the two values of resistance characteristic of the two folded states (Figure 6E). Notably, the resistance values faithfully tracked the deformation at all evaluated frequencies (0.25, 0.5, 1, and 2 Hz). These results proved the effectiveness of the proposed material in tracking bending deformations in a reversible fashion, thus evidencing its potential as element for motion sensing.

### Hydrophobization of Bionanocomposites Surfaces

**Figure 7:**
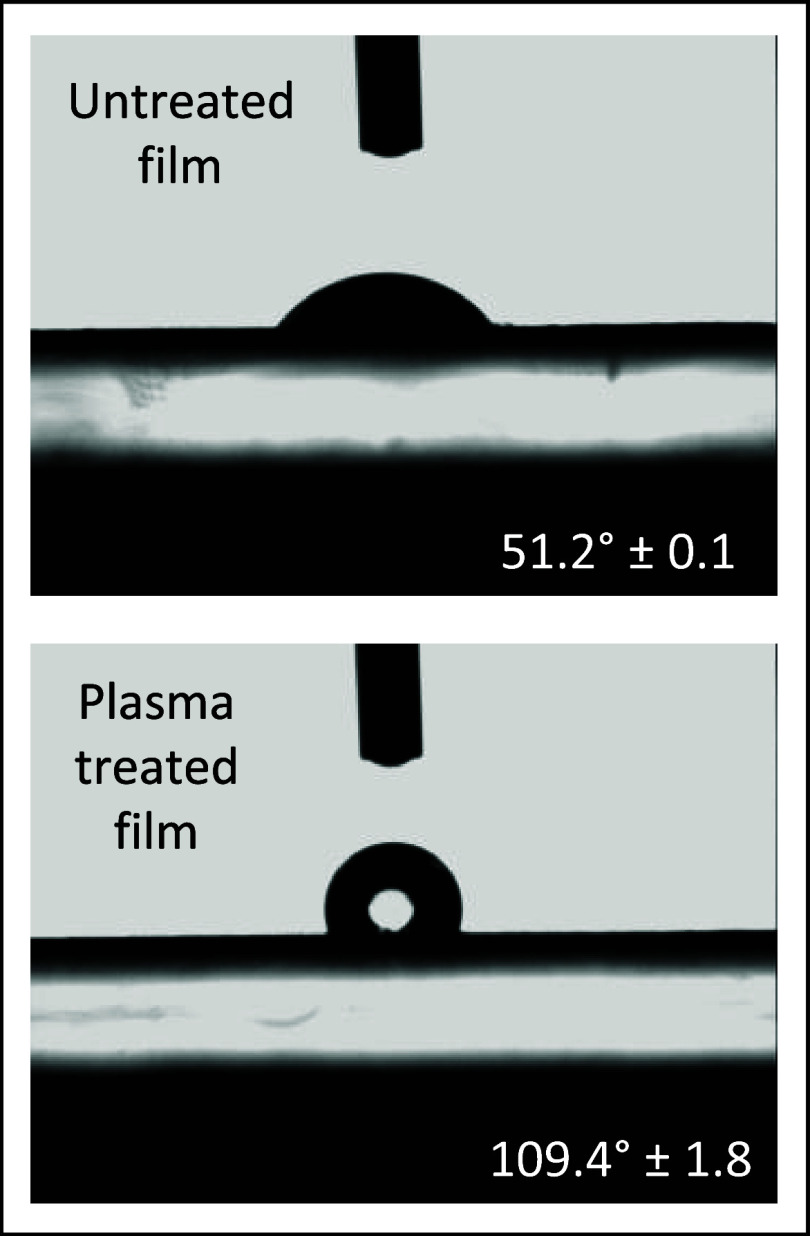
High surface hydrophobicity of BSF protein nanocomposites after LPCP treatment. Static water contact angle (WCA) pictures and calculated angles for the 25 phm CCB/SP-containing bionanocomposite were either submitted or not to the environmentally sustainable hydrophobizing cold plasma treatment.

The high hydrophilicity of biobased materials, including protein-based materials, is a feature that can result in high sensitivity of material performance in humid environments, i.e., most application conditions.^72^ The change in the material’s intrinsic conductivity, caused by interpenetration of water molecules in the composite structure, was exploited for instance to create humidity sensors for flexible electronics.^17,73−75^ The ability to make protein-based materials hydrophobic could broaden their range of applications, particularly in the field of flexible electronics. In this work, a hydrophobic coating of polymerized hexamethyldisiloxane (HMDSO) was applied on a protein substrate through a low-pressure cold plasma (LPCP) treatment, as detailed in the Experimental Section. LPCP is indeed known as a dry and eco-sustainable method for modifying material surfaces without altering their bulk properties.^76−78^ Here, the thickness of the deposited layer was measured to be 468.8 ± 6.7 nm. After the treatment, the surface hydrophobicity of all samples was significantly increased, enabling to achieve static water contact angles (WCA) up to 120° (Figure 7). Furthermore, higher WCA values were found for composites enriched with CCB, either with or without functionalization (Figure S10). The high values of WCA obtained after the treatment prove the effectiveness of LPCP to impart high hydrophobicity to the here presented bionanocomposites. Together with the above-mentioned sustainability advantages, it can be reasonably assumed that LPCP technology can be easily used in general on protein-based materials, thereby enhancing their water stability and, hence, their applicability.

## Conclusions

Proteins derived from the BSF insect, utilized as bioconverter of the OFMSW, were used for the first time to create a (bio)degradable, electrically conductive bionanocomposite suitable for disposable flexible electronic devices. The use of BSF proteins for GFE appears to offer remarkable advantages: the valorization of a product deriving from an efficient treatment of OFMSW, the use of a protein-rich feedstock not in competition with the food chain, and which requires few natural resources (land, water) and energy. When dispersed in alkaline water, BSF proteins were found able to undergo supramolecular ordered aggregation, creating physical networks suitable as matrix of composite materials without requiring much energy or toxic elements, as organic solvents, cross-linkers, or oil-based polymers, thereby adhering to green chemistry principles. The bionanocomposites reached electrical conductivity values as high as 0.9 × 10^–2^ S/cm at relatively low filler volumetric fractions (8% v/v), with ranges that well reflect those required by electromagnetic shielding and antistatic applications, as well as those for circuit’s resistive elements in flexible electro-mechanical/chemical sensors. In this regard, the bionanocomposite displayed reversible sensing properties toward bending deformations, demonstrating potential applicability in smart clothing for human motion sensing. Notably, high values of WCA were achieved by depositing an ultrathin layer of polysiloxane via an environmentally friendly plasma treatment, further enhancing the applicability of these materials in environments characterized by relatively high humidity.

The interest and advantages of the nanocomposites reported here seem to be based on the unprecedented combination of many aspects: the use of proteins from OFMSW, as commented above, and of commercially available CB, the obtainment of performances in line with the best reported in the literature, the high surface hydrophobicity, and the biodegradability of the composite matrix.

The composites herein developed thus lay the groundwork for developing circular materials from the bioconversion of the OFMSW, contributing to close a novel circular economy model for its full valorization while boosting sustainable approaches in the electronics sector.

## Experimental Section/Methods

### Materials

Protein extracts were from the pupal stage of BSF, reared on surrogate samples of OFMSW.^38^ Extracts were obtained in the framework of a recent study published by some of the authors.^39^ The composition of the OFMSW substrate and protein extraction methods were reported in the same study.^39^ Conductive Carbon Black (CCB) was an ENSACO 360G instrument from Imerys. Carboxymethylcellulose (CMC) was obtained from Fluka BioChemika. Unless otherwise specified, the remaining chemicals were from Merck/Sigma-Aldrich.

### Characterization of BSF Protein Extracts

#### Proteomic Analysis of BSF Pupae Protein Extracts

The quantification of soluble protein content in BSF protein extracts was conducted with the bicinchoninic acid assay (BCA) according to the manufacturer’s specifics (Pierce BCA Protein Assay kit, Thermo Fisher Scientific). Measurements were carried out on 5 different biological samples. The pool of protein molecular weights (MW) within the extracts was resolved through sodium dodecyl sulfate-polyacrylamide gel electrophoresis (SDS-PAGE). Dried protein extracts were resuspended Milli-Q water at pH 12 and mixed with Laemmli buffer 2× as reported in Text S1. Gel preparation and electrophoresis procedures adhered to standardized protocols.^79^ Three samples from three different biological replicates were subjected to the analysis. Protein identification was accomplished through nanoliquid chromatography–mass spectrometry (nLC-MS/MS) analyses, performed with a LTQ-XL spectrometer (Thermo). The more intense bands of SDS-PAGE gels (i.e., 75, 50, 30, 20 kDa) were analyzed for three different biological samples. Gel bands were processed in accordance with established protocols as detailed in prior publications.^80^ For each gel band, the cumulative mass spectrometry data for the three biological replicates underwent analysis utilizing the Mascot search engine (Version 2.3.01), coupled with the Proteome Discoverer software (Version 1.2.0 Thermo), and referencing the UniProtKB/SwissProt protein database (Uniprot_InsectaRev Insecta_Reviewed/Current/InsectaReviewed, encompassing 10974 sequences and 5363805 residues).

#### Protein Aggregation Monitoring

The aggregation propensity of BSF pupae proteins was monitored through ThioflavinT (ThT) fluorescence spectroscopy. 2 mM ThT stock solutions were prepared dissolving the ThT powder in Milli-Q water. The suspension was next filtered with a 0.22 μm syringe filter to remove the insoluble matter. The ThT working solution (20 μM) was obtained by diluting the ThT stock solution 100 times with Milli-Q water. For aggregation experiments, 10 μL of BSF protein suspensions (5% w/v in NaOH 0.1 M) was taken out and mixed with 190 μL of ThT working solution in a 96-well microplate. The ThT fluorescence intensity of tested samples was measured by a Synergy H1 reader (BioTek, Winooski, VT), λ_ex_ = 450 nm; λ_em_ = 490 nm, at 25 °C.

### Preparation and Characterization of SP and CCB/SP Adduct

#### Synthesis of 2-(2,5-Dimethyl-1*H*-pyrrol-1-yl)-1,3-propanediol (Serinol Pyrrole, SP)

The synthesis of SP was conducted as described in previous research.^64^ The reader is referred to Text S2 for a detailed description of the procedure utilized in this work.

#### Functionalization of CCB with SP

Functionalization of CCB was performed according to a standardized procedure for sp^2^ carbon allotropes functionalization with pyrrole compounds, as detailed in previous research.^61^ The reader is referred to Text S3 for a detailed description of the synthesis procedure utilized for this work.

#### Thermogravimetric Analysis of CCB and CCB/SP

The degree of functionalization (DoF) of CCB/SP and functionalization yield (FY) were evaluated by means of thermogravimetric analyses (TGA). Mass losses were measured as a function of time and temperature on an SDT Q600 V20.9 Build 20 TGA/DTA Instruments using the standard method ISO9924-1. The detailed procedure and equations for the calculation of DoF and FY are reported in Text S5.

#### X-ray Diffraction Analysis of CCB and CCB/SP

Crystalline patterns of CCB and the CCB/SP adduct were investigated by means of wide-angle X-ray powder diffraction (WAXRD). XRD patterns were obtained in reflection, with an automatic Bruker D8 Advance diffractometer with nickel filtered Cu–Kα radiation. Patterns were recorded in 4–90° as the 2θ range, with 2θ being the peak diffraction angle.

#### Determination of Hansen Solubility Parameters

The calculation of the Hansen Surface Parameters (HSP) for CCB and CCB/SP samples was performed by applying the Hansen Solubility Sphere representation of miscibility for sp^2^ carbon allotropes, as reported in previous research.^62^ Obtained visual data were acquired and organized as in Table 1, which served as input for the Matlab algorithm. The fitting sphere algorithm was adapted from the code by F. Gharagheizi and solved in Matlab environment using the Nelder–Mead simplex algorithm.^81^

### Preparation and Characterization of Bionanocomposite Films

#### Preparation of Bionanocomposite Films

Film preparation was performed through a wet method with water as the only solvent. The conductive filler (i.e., CCB or CCB/SP) was weighed in a 10 mL becher in quantities ranging from 0 up to 75 mg, depending on the amount of desired filler. 2.9 mL of Milli-Q water was then added, followed by 1.6 mL of a CMC 3% w/v solution. 500 μL of NaOH 1 M was added, raising pH up to 12. The mixture was stirred for 5 min, then tip-sonicated (Branson SFX550, 550W, 20 kHz, 20% amplitude) for 1 min, followed by a 1 min pause. The cycle was repeated four times. 250 mg of BSF protein extracts were next weighed and added to the suspension, to give a 5%w/v protein concentration. 125 mg of glycerol was added immediately after. The mixture was stirred for 10 additional minutes, cast in a squared PDMS mold (40 mm × 40 mm), and left to dry at room temperature (RT, 20 ± 2 °C) for 2 days. Details on the absolute and relative amount of ingredients used for each formulation are reported in Table S4. Films were stored in sealed plastic bags in a dark environment until further characterizations, unless otherwise specified.

#### Water Uptake Tests and Determination of Film–Water Interaction Parameters

Water uptake tests were conducted by submerging films in Milli-Q water at the RT (20 ± 2 °C) after overnight conditioning in an oven at 80 °C. A detailed description of the test, calculated parameters, and equations is reported in Text S8. The obtained gravimetric data were implemented to solve the Kraus equation,^65^ as applied elsewhere.^66,67^ The mathematical model and equations behind the calculation of the Kraus constant are reported in Text S9.

#### Degradability Experiments

For degradability experiments, films were first conditioned for 24 h at 80 °C in a ventilated oven. Degradability experiments were conducted by exposing films of standardized dimensions (5 mm × 20 mm) in a 1 M HCl water solution kept at 90 °C under magnetic stirring and reflux condenser. (Bio)degradability experiments were conducted by exposing the same films to a 1% w/v water-pepsin solution (pH 4) kept at 36 °C under magnetic stirring, as reported in previous research.^17^ For reference, films were exposed to the same water solution (pH 4 at 36 °C) without pepsin. The degradability of the films was visually examined by monitoring structure integrity and the creation of black suspensions generated from the release of CCB due to matrix degradation. Powders were recovered from suspensions by centrifugation, resuspension in acetone, and acetone evaporation.

#### Preparation and Characterization of Low-Pressure Plasma Hydrophobic Coatings

Plasma-polymerized hexamethyldisiloxane (HMDSO) coatings were deposited on previously prepared protein-based composites in low-pressure plasma equipment, with a stainless-steel chamber reaction with a volume of 100 L. The plasma reactor was operating at a fixed frequency of 13.56 MHz. The gas feed composition is 100% HMDSO. An α-step 500 profilometer (Tencor Instruments) was utilized for thickness measurements (resolution: 3 ± 0.3 nm), utilizing a mask to create jumps representative of the coating thickness.

Static water contact angle (WCA) tests were performed on a Contact Angle System OCA 15plus instrument (Dataphysics). 2 μL drops were dispensed through a 500 μL Hamilton syringe on the interested surface at a rate of 1 μL/s. For WCA quantification, spherical model contour analyses were performed. Tests were carried out in triplicate for each tested film sample.

#### Transmission Electron Microscopy (TEM)

The digital image analysis of filler dispersion through TEM was carried out following three steps: material slicing, image acquisition, and visual analysis. A cryo-ultramicrotome (type: Leica EM FC 6, Leica Microsystem, Wetzlar, Germany) using a diamond knife (type: Diatome 35°) at a temperature of −80 °C was used to produce sections with a width of approximately 100 μm and a thickness of approximately 100 nm. The preparation was carried out using the wet cutting method, in which the sections are floated on a mixture of dimethyl sulfoxide (DMSO) and water (50/50), and next transferred, using a loop, to a 400 mesh copper grid coated with poly(vinyl formal) (Plano). TEM analysis was performed with a TEM, LIBRA 120, Zeiss, Oberkochen (Baden-Württemberg, Germany), with an acceleration voltage of 120 kV.

#### Electrical Conductivity Measurements

Electrical conductivity (σ) of films was assessed by measuring the electrical volumetric (thickness-through) resistance (*R*_v_) by means of a Keysight Technologies 34450A Digital Multimeter (Keysight Technologies, Milan, Italy). The films were first conditioned at 30 °C in a ventilated oven for at least 2 days. A detailed description of the setup is reported in Text S10. Dynamic bending resistance measurements were collected with the same instrument (Keysight Technologies 34450A Digital Multimeter) using a four-point probe configuration. Four-wire resistance measurements were recorded by the Keysight BenchVue software (V 1.03) as a function of time while applying bending/stretching cycles at different frequencies (0.25, 0.50, 1, and 2 Hz). Time resolution throughout the measurement was set to 256 μs. The obtained resistance vs time trace was analyzed by OriginPro 2018.

## Supporting Information Available

Additional experimental details, materials, and methods, including photographs of experimental setup (PDF)
Raw data tables from nLC-MS/MS analysis—MASCOT search (XLSX)
