# Supplementary material for: Electroconductive Bionanocomposites from Black Soldier Fly Proteins for Green Flexible Electronics
Source: ACS Sustain Chem Eng. 2025 Feb 3;13(6):2388–400. doi: 10.1021/acssuschemeng.4c08242 (PMC11837284; doi:10.1021/acssuschemeng.4c08242)
Supplement: Supplementary file 1 — sc4c08242_si_001.pdf [file sc4c08242_si_001.pdf]

Electroconductive Bionanocomposites from Black Soldier Fly Proteins for Green Flexible Electronics

Edoardo Testa,[1] Vincenzina Barbera,[1] Elisa Fasoli,[1] Ulrich Giese,[2] Maria Rosaria Belviso,[3] Pasqua Rossini,[3] Daniele Bruno,[4] Gianluca Tettamanti,[4,5] Marco Orlando,[4] Gianluca Molla,[4] Morena Casartelli,[5,6] Maurizio Galimberti [1]*

[1] Politecnico di Milano, Department of Chemistry, Materials and Chemical Engineering "G. Natta", Via Mancinelli 7, 20131 Milano, Italy

[2] Deutsches Institut für Kautschuktechnologie e. V., Eupener Straße 33, 30519 Hannover, Germany

[3] Plasmapps Srl, Via VVF Caduti in Servizio 14, Zona artigianale Modugno, 70126 Bari, Italy

[4] Università degli Studi dell'Insubria, Department of Biotechnology and Life Sciences, Via J. H. Dunant 3, 21100 Varese, Italy

[5] Interuniversity Center for Studies on Bioinspired Agro-environmental Technology (BAT Center), Università di Napoli Federico II, Piazza Carlo di Borbone 1, 80055 Portici, Italy

[6] Università degli Studi di Milano, Department of Biosciences, Via Celoria 26, 20133 Milano, Italy

*Corresponding author:

Maurizio Galimberti (maurizio.galimberti@polimi.it)

Keywords: Proteins, Nanocomposite, Black Soldier Fly, Organic Waste, Flexible electronics

Contents of Supporting Information

This document contains a total of 24 pages, including 6 tables, 10 figures, 11 supplementary texts and references.

Contents

Supplementary Tables

Table S.1. Raw data tables from nLC-MS/MS analysis, performed by MASCOT search engine (provided as separate .xlsx file).

Table S.2. Table of proteins identified by nLC-MS/MS analysis, reports Mascot score, molecular weight and number of identified peptides. Band position and colors correspond to SDS-PAGE bands depicted in Figure S.1.

Table S.3. Mass losses from thermogravimetric curves in Figure 3A for CCB and the CCB/SP adduct.

Table S.4. Bionanocomposites film composition by weight (absolute and relative weight compositions) and volume.

Table S.5. Sol/Gel fraction data for the two tested sets of bio-nanocomposites as a function of their filler contents.

Table S.6 Collection of current nanocomposite materials and their performances as components for flexible electronics applications.

Supplementary Figures

Figure S.1. SDS-PAGE analysis of BSF pupae protein extracts dispersed in Milli-Q water as described in Text S.1. The three protein profiles represent the three different biological replicates. Colored parenthesis on the densitogram are representative of the gel bands analysed by nLC-MS/MS (see Table S.1 and S.2).

Figure S.2. (A) Fluorescence spectra of BSF protein extract solutions (2.5 mg/mL). Reported spectra were recorded for BSF protein suspension at pH 12 with ThT (continuous blue line) and without ThT (dashed blue line). Reference controls (black lines) were prepared in the same conditions without adding BSF protein extract to Milli-Q water. (B) Development of intermolecular β-sheet interactions monitored through ThT fluorescence assay. The graph reports the ThT fluorescence intensity at 490 nm as a function of time for diluted BSF protein extract solutions at pH 12 (blue lines) and pH 2 (orange lines). Dashed lines are representative of the same suspensions without ThT.

Figure S.3. Step-by-step procedure for the obtainment of CCB/SP. (B) Chemical species involved in the production of the CCB/SP adduct.

Figure S.4. XRD diffractograms of CCB (black curve) and CCB/SP (red curve). Main reflections are characteristic of graphene layers *in-plane* order (100) (i.e., repeating units along the graphene layer) and *out-of-plane* order (002) (i.e. stacked graphene layers, with characteristic distance of 0.34nm).

Figure S.5. Precipitation stability test. The test was conducted against the following solvents: Water, Acetone, Ethyl Acetate, Hexane, Chloroform, Dichloromethane, Methanol, 1-Buthanol, Tetrahydrofuran and 2-Propanol. CCB or CCB/SP suspensions at 1mg/ml concentration were prepared for each solvent and next sonicated in a bath sonicator for 20 minutes. Pictures were taken after 1 day to allow settling where present. The stability was monitored for 1 month to assess further changes in time: the situation remained as stable as after 1 day.

Figure S.6. Hansen spheres of CCB (I) and CCB/SP (II).

Figure S.7. Volumetric variation (ΔV%) as a function of the filler content for both set of bio-nanocomposite films (black columns for CCB and red columns for CCB/SP).

Figure S.8. Film solubility (FS%) as a function of the filler content for both sets of bio-nanocomposite films (black columns for CCB and red columns for CCB/SP).

Figure S.9. Degradation of the BSF protein nanocomposite and recovery of CCB. (I) Starting film sample; (II) the film is degraded in 1M HCl or 1% pepsin-water solutions; (III) CCB is unstable in water solutions and

rapidly precipitate after a centrifugation step; (IV) CCB was recovered by resuspension in acetone and subsequent evaporation of the solvent.

Figure S.10. Static water contact angle (WCA) pictures and measurements for 25 phm CCB and CCB/SP-containing nanocomposites.

Supplementary Texts

Text S.1. Preparation of BSF pupae protein extracts in Milli-Q water for SDS-PAGE analysis.

Text S.2. Synthesis of 2-(2,5-dimethyl-1H-pyrrol-1-yl)-1,3-propanediol (Serinol pyrrole, SP).

Text S.3. Functionalization of CCB with SP.

Text S.4. Mechanisms underlying the functionalization of CCB with SP.

Text S.5. Detailed process for TGA analysis and calculation of DoF and FY.

Text S.6. Interpretation of thermograms for CCB and CCB/SP.

Text S.7. Details about the formulation of BSF protein nanocomposite films

Text S.8. Experimental procedure for swelling tests and determination of water interaction parameters.

Text S.9. Determination of the Kraus constant from the Kraus model.

Text S.10. Experimental setup for the measurement of R_V.

Text S.11. Percolation theory model applied to presented materials.

Text S.1. Preparation of BSF pupae protein extracts in Milli-Q water for SDS-PAGE analysis.

Dried protein extracts were resuspended in Milli-Q water to a final concentration of 5 mg/mL, and their pH adjusted dropwise to 12 using 1M NaOH. Subsequently, the samples were incubated at 80°C for 20 minutes. A 10 µL aliquot of the 5 mg/mL protein extract solution (corresponding to 50 µg of proteins) was then combined with 10 µL of Laemmli buffer 2X (62.5 mM Tris–HCl, pH 6.8, 2% SDS, 25% glycerol, and 0.01% bromophenol blue, BioRad) and loaded into the gel.

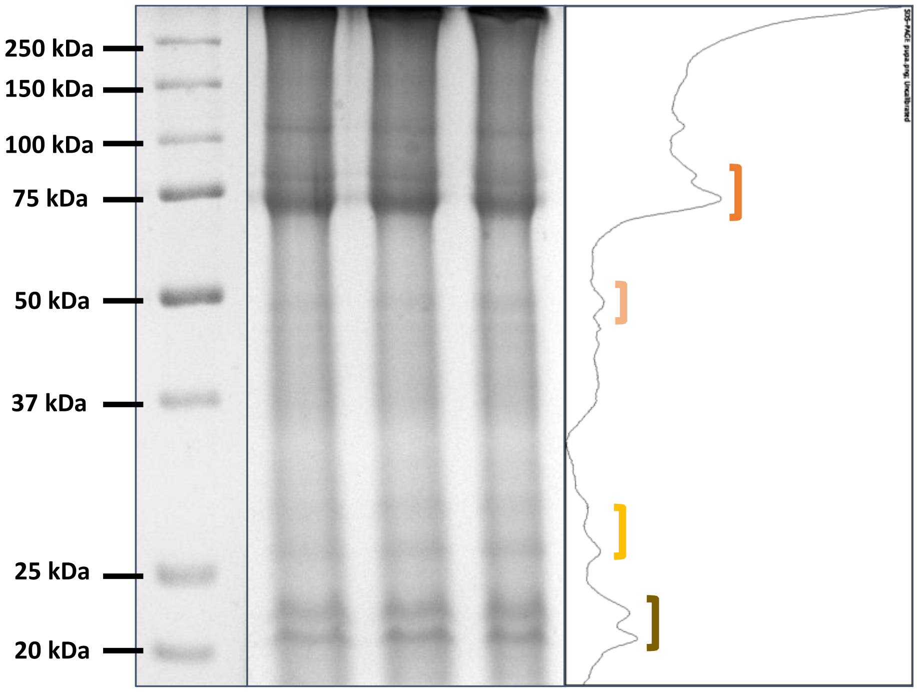

Figure S.1. SDS-PAGE analysis of BSF pupae protein extracts dispersed in Milli-Q water as described in Text S.1. The three protein profiles represent the three different biological replicates. Colored parenthesis on the densitogram are representative of the gel bands analysed by nLC-MS/MS (see Table S.1 and S.2).

Table S.1. Raw data tables from nLC-MS/MS analysis, performed by MASCOT search engine (provided as separate .xlsx file).

Table S.2. Table of proteins identified by nLC-MS/MS analysis, reports Mascot score, molecular weight and number of identified peptides. Band position and colors correspond to SDS-PAGE bands depicted in Figure S.1.

Accession Number	Protein Name	Mascot Score	MW (Da)	N° peptides	Band position
P05661-2	Isoform BDBBA of Myosin heavy chain, muscle OS=Drosophila melanogaster OX=7227 GN=Mhc	287	225299	15	75 kDa
P05661-2	Isoform BDBBA of Myosin heavy chain, muscle OS=Drosophila melanogaster OX=7227 GN=Mhc	391	225299	13	50 kDa
Q05825	ATP synthase subunit beta, mitochondrial OS=Drosophila melanogaster OX=7227 GN=ATPsynbeta	759	54074	11	50 kDa
P06603	Tubulin alpha-1 chain OS=Drosophila melanogaster OX=7227 GN=alphaTub84B PE=1 SV=1	216	50561	3	50 kDa
P19351-12	Isoform 12 of Troponin T, skeletal muscle OS=Drosophila melanogaster OX=7227 GN=up	178	46513	7	50 kDa
P05661	Myosin heavy chain, muscle OS=Drosophila melanogaster OX=7227 GN=Mhc PE=1 SV=4	211	225411	5	30 kDa
Q9XZ71	Troponin T OS=Periplaneta americana OX=6978 GN=TNT PE=2 SV=1	107	45880	4	30 kDa
Q95PM9	Arginine kinase OS=Plodia interpunctella OX=58824 GN=ARGK PE=1 SV=1	97	40139	2	30 kDa
P48610	Arginine kinase OS=Drosophila melanogaster OX=7227 GN=Argk PE=2 SV=2	93	40126	2	30 kDa
Q1HPQ0	Tropomyosin-2 OS=Bombyx mori OX=7091 PE=1 SV=1	173	32823	3	30 kDa
Q1HPU0	Tropomyosin-1 OS=Bombyx mori OX=7091 PE=1 SV=1	156	32603	2	30 kDa
P36188	Troponin I OS=Drosophila melanogaster OX=7227 GN=wupA PE=2 SV=3	61	30234	4	30 kDa
P05661	Myosin heavy chain, muscle OS=Drosophila melanogaster OX=7227 GN=Mhc PE=1 SV=4	97	225411	4	20 kDa
P07836	Actin, muscle-type A1 OS=Bombyx mori OX=7091 PE=3 SV=1	101	42248	3	20 kDa
P14318	Muscle-specific protein 20 OS=Drosophila melanogaster OX=7227 GN=Mp20 PE=2 SV=2	285	20292	2	20 kDa
P47949	Troponin C, isoform 3 OS=Drosophila melanogaster OX=7227 GN=TpnC73F PE=2 SV=2	277	17742	4	20 kDa
P45594	Cofilin/actin-depolymerizing factor homolog OS=Drosophila melanogaster OX=7227 GN=tsr PE=1 SV=1	66	17428	2	20 kDa

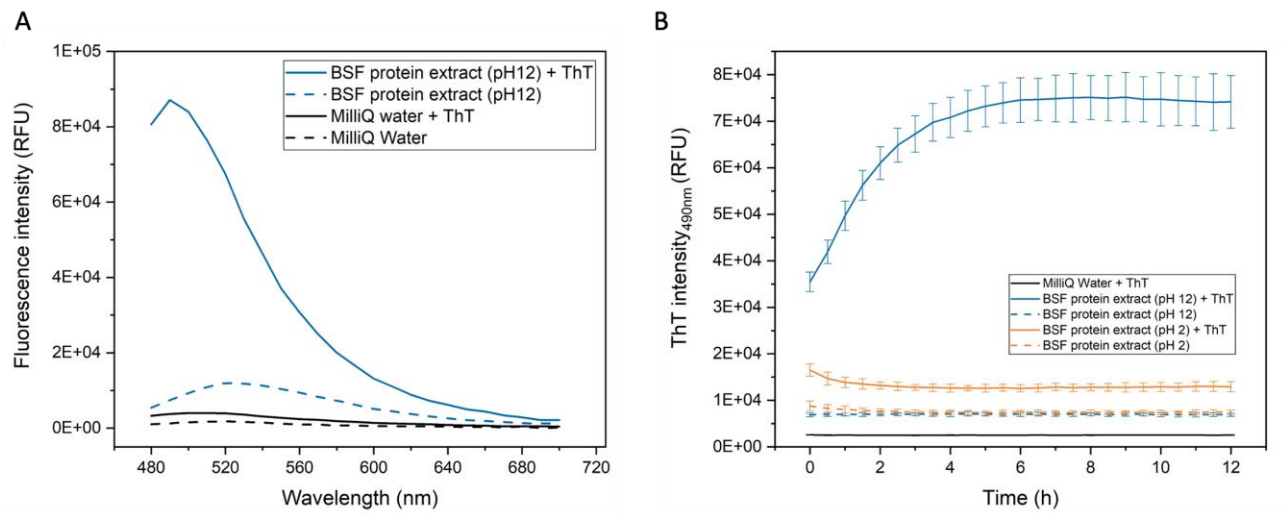

Figure S.2. (A) Fluorescence spectra of BSF protein extract solutions (2.5 mg/mL). Reported spectra were recorded for BSF protein suspension at pH 12 with ThT (continuous blue line) and without ThT (dashed blue line). Reference controls (black lines) were prepared in the same conditions without adding BSF protein extract to Milli-Q water. (B) Development of intermolecular β-sheet interactions monitored through ThT fluorescence assay. The graph reports the ThT fluorescence intensity at 490 nm as a function of time for diluted BSF protein extract solutions at pH 12 (blue lines) and pH 2 (orange lines). Dashed lines are representative of the same suspensions without ThT.

Text S.2. Synthesis of 2-(2,5-dimethyl-1H-pyrrol-1-yl)-1,3-propanediol (Serinol pyrrole, SP).

6.46 g (0.071 mol) of serinol (powder, MW = 91.11 g/mol) were poured inside a 100-mL round bottomed flask equipped with a magnetic stirrer and heated at 155°C for 15 minutes, then 8.10 g (0.071 mol) of 2,5-hexanedione (Liquid, MW = 114.14 g/mol) was added dropwise to the liquid serinol. The obtained solution was stirred for 2 hours in the same flask equipped with a reflux condenser. The condenser was next removed, and the solution kept heated for 30 minutes. After this time, reaction was stopped by cooling down the vessel to room temperature (R.T., 20 ± 2 °C). Pure product 2-(2,5-dimethyl-1H-pyrrol-1-yl)-propane-1,3-diol (SP) was obtained as a dark amber viscous liquid.

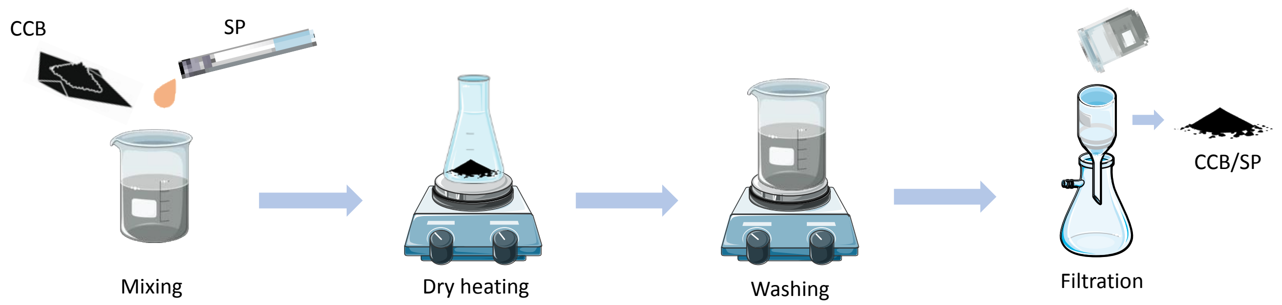

Figure S.3. Step-by-step procedure for the obtainment of CCB/SP.

Text S.3. Functionalization of CCB with SP.

2.00 g of CCB were poured into a 100 mL round bottomed flask, then 50 mL of acetone were added. Subsequently, the suspension was sonicated through a bath sonicator apparatus (Ultrasonic Cleaner 2200 S3, Sonica) for 10 minutes. 0.47 g of SP were next weighed in a glass vial, solubilized in 8 mL of acetone, and poured into the CCB/acetone suspension. The mixture was eventually homogenized by means of a bath sonicator apparatus for 10 additional minutes. Acetone was next removed in a rotavapor machine (Rotavapor RII, BUCHI Switzerland) at 40°C and 300 mbar. The dried sample was transferred in a 250 mL two-neck round-bottom flask. The flask was equipped with a magnetic stirrer and positioned in an oil bath at 180°C under stirring (300 rpm). Furthermore, an air flux was introduced from the secondary neck of the flask while on the main neck an air condenser with sintered septum is added. The system was left under stirring for 2 hours. The reaction was therefore carried out neat, without solvents. A Soxhlet extraction with acetone was eventually performed on the obtained powder to remove the fraction of unreacted SP (T = 90°C, t = 16h).

Text S.4. Mechanisms underlying the functionalization of CCB with SP.

The functionalization of CCB occurred through a domino reaction. The carbon substrate induced the oxidation of SP through a carbo-catalyzed process, leading to the formation of an aldehydic group on the grafted SP. A Diels-Alder cyclo-addition reaction occurs between the sp^2 carbon allotrope and SP, where SP acts as the dienophile, and the aromatic ring of the graphene layer serves as the diene.

Text S.5. Detailed process for TGA analysis and calculation of DoF and FY.

The procedure, performed on 10 mg of CCB or CCB/SP samples, consisted in a heating ramp (10°C/min) from 30 up to 300°C, followed by 10 minutes isotherm at 300°C, then another heating ramp (20°C/min) up to 550°C and, subsequently, a 15 minutes isotherm at 550°C; a final heating ramp (10°C/min) up to 900°C was followed by a 20 minutes isotherm to the end of the experiment. The whole procedure was performed in N_2 atmosphere except for the last 20 minutes, in which the gas in the chamber was switched to O_2. The DoF and the FY were calculated through Equations (1) and (2) as described elsewhere [1].

$$DoF\ (\%) = [CCB/SP\ wt.loss(\%)_{150-900°C} - CCB\ wt.loss(\%)_{150-900°C}] \quad (1)$$

$$FY(\%) = \frac{DoF(\%)}{(SP\ wt.(mg)_{\ exp})/(SP\ wt.(mg)_{\ exp} + CCB\ wt.(mg)_{exp})} \times 100 \quad (2)$$

Text S.6. Interpretation of thermograms for CCB and CCB/SP.

The initial weight loss (<150°C) was attributed to the removal of excess water from the sample. The thermal decomposition of CCB/SP at higher temperatures exhibited two primary steps, both within the temperature range from 150 °C to 900 °C. These steps were associated with the decomposition of alkenyl-, oxygen-, and nitrogen-containing functional groups. The quantification of SP content within the adduct was determined by assessing the mass loss within this range of temperatures. The subsequent and final decomposition phase occurred at temperatures exceeding 900 °C and was attributed to the combustion of the graphitic structure after reacting with atmospheric oxygen.

Table S.3. Mass losses from thermogravimetric curves in Figure 3A for CCB and the CCB/SP adduct.

	**Mass loss (%)**		
	**T<150°C**	**150°C<T<900°C**	**T>900°C**
**CCB**	0.5	1.1	98.4
**CCB/SP**	1.4	12.1	86.5

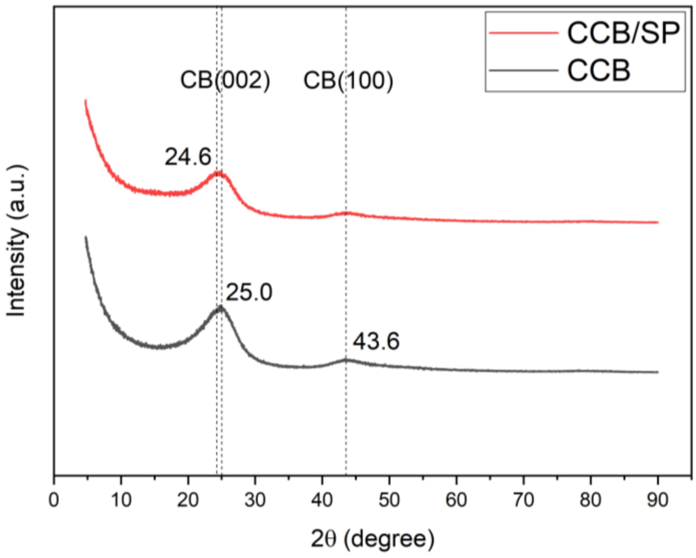

Figure S.4 XRD diffractograms of CCB (black curve) and CCB/SP (red curve). Main reflections are characteristic of graphene layers *in-plane* order (100) (i.e., repeating units along the graphene layer) and *out-of-plane* order (002) (i.e. stacked graphene layers, with characteristic distance of 0.34nm).

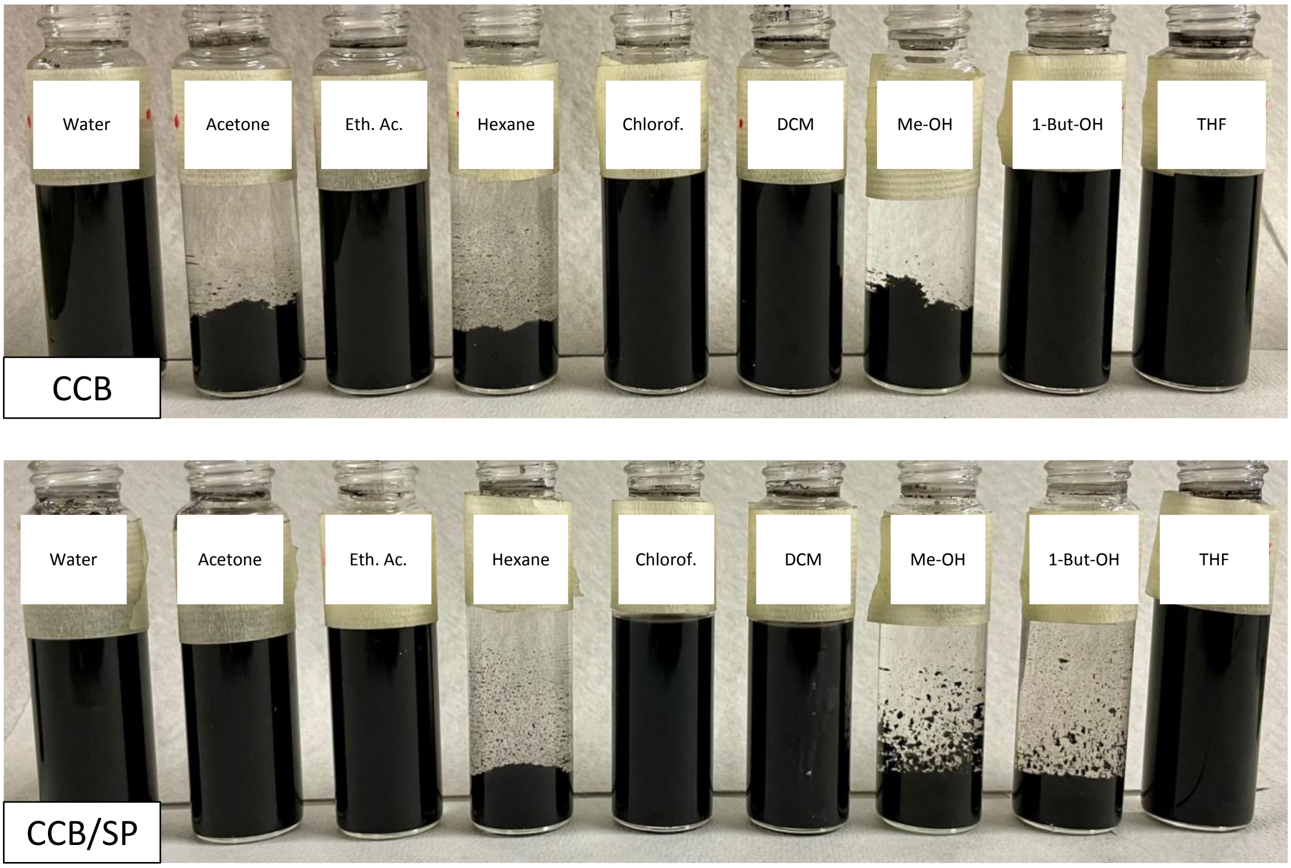

Figure S.5. Precipitation stability test. The test was conducted against the following solvents: Water, Acetone, Ethyl Acetate, Hexane, Chloroform, Dichloromethane, Methanol, 1-Buthanol, Tetrahydrofuran and 2-Propanol. CCB or CCB/SP suspensions at 1mg/ml concentration were prepared for each solvent and next sonicated in a bath sonicator for 20 minutes. Pictures were taken after 1 day to allow settling where present. The stability was monitored for 1 month to assess further changes in time: the situation remained as stable as after 1 day.

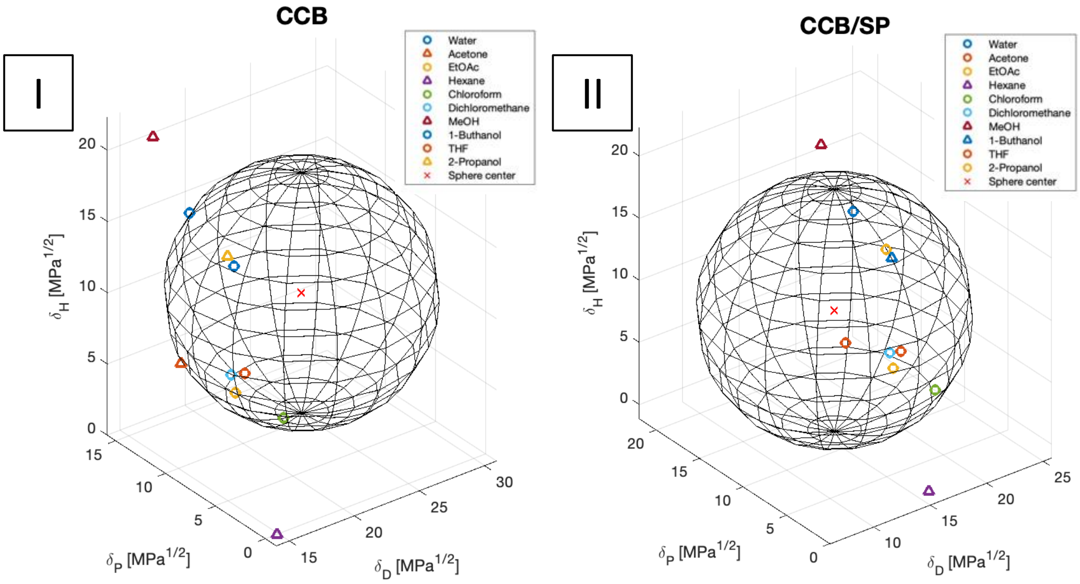

Figure S.6. Hansen spheres of CCB (I) and CCB/SP (II).

Text S.7. Details about the formulation of BSF protein nanocomposite films.

In the initial phase of this study, the formation of homogeneous aqueous dispersions containing proteins from BSF and CCB was tested. It was observed that functionalization with SP alone was insufficient to ensure long-term stability against precipitation of the filler, yielding uneven bionanocomposite films. To address this issue, carboxymethylcellulose (CMC) was added, thereby enhancing the stability of the CCB dispersions and homogeneity of the bionanocomposites. This finding is in line with previous research,[2] wherein polyvinylpyrrolidone (PVP) was used as a polymeric stabilizer for graphene platelets to enhance their dispersion stability. A sonication step was added to break down macroscopical aggregates of CCB.

The protein extracts were added to the aqueous mixture of CCB and CMC after the ultrasonication step, to avoid the potential disruption of polypeptide chains caused by the high thermal and mechanical energy inherent in the sonication process.

Glycerol was added up to 50% w/w on BSF protein extract weight to ensure high film's flexibility, according to previous works findings.[3] Lower glycerol contents (< 40%) yielded brittle films.

Table S.4. Bionanocomposites film composition by weight (absolute and relative weight compositions) and volume.

	Absolute weight in formulation [mg]			
**ID**	**CCB**	**CMC**	**Glycerol**	**BSF pupae protein extract**
**0 phm[a] CCB**	0	50	125	250
**5 phm CCB**	15	50	125	250
**10 phm CCB**	30	50	125	250
**12 phm CCB**	36	50	125	250
**15 phm CCB**	45	50	125	250
**20 phm CCB**	60	50	125	250
**25 phm CCB**	75	50	125	250
	**CCB/SP**	**CMC**	**Glycerol**	**BSF pupae protein extract**
**5 phm CCB/SP**	15 (1.8) [b]	50	125	250
**10 phm CCB/SP**	30 (3.6) [b]	50	125	250
**12 phm CCB/SP**	36 (4.3) [b]	50	125	250
**15 phm CCB/SP**	45 (5.4) [b]	50	125	250
**20 phm CCB/SP**	60 (7.2) [b]	50	125	250
**25 phm CCB/SP**	75 (9.0) [b]	50	125	250

[a] **phm**: per hundred matrix, matrix = CMC + BSF proteins

	Relative contents in final composites [%] *			
**ID**	**CCB**	**CMC**	**Glycerol**	**BSF pupae protein extract**
**0 phm CCB**	0	12	29	59
**5 phm CCB**	3	11	28	57
**10 phm CCB**	7	11	27	55
**12 phm CCB**	8	11	27	54
**15 phm CCB**	10	11	27	53
**20 phm CCB**	12	10	26	52
**25 phm CCB**	15	10	25	50
	**CCB/SP**	**CMC**	**Glycerol**	**BSF pupae protein extract**
**5 phm CCB/SP**	3 (0.36) [a]	11	28	57
**10 phm CCB/SP**	7 (0.84) [a]	11	27	55
**12 phm CCB/SP**	8 (0.96) [a]	11	27	54
**15 phm CCB/SP**	10 (1.20) [a]	11	27	53
**20 phm CCB/SP**	12 (1.44) [a]	10	26	52
**25 phm CCB/SP**	15 (1.80) [a]	10	25	50

a numbers within parentheses show the content of SP on the total weight of the composite (DoF (%) = 11% w/w)

* Determined considering complete dehydration of the films.

	Volumetric fraction in final composites [ϕ]*			
**ID**	**CCB** [a]	**CMC** [b]	**Glycerol** [c]	**BSF pupae protein extract** [d]
**0**	0,00	0,08	0,24	0,68
**5 phm CCB**	0,02	0,08	0,24	0,67
**10 phm CCB**	0,04	0,07	0,23	0,65
**12 phm CCB**	0,05	0,07	0,23	0,65
**15 phm CCB**	0,06	0,07	0,23	0,64
**20 phm CCB**	0,08	0,07	0,22	0,63
**25 phm CCB**	0,09	0,07	0,22	0,62
	**CCB ****	**CMC**	**Glycerol**	**BSF pupae protein extract**
**5 phm CCB/SP**	0,02	0,08	0,24	0,67
**10 phm CCB/SP**	0,03	0,07	0,23	0,66
**12 phm CCB/SP**	0,04	0,07	0,23	0,65
**15 phm CCB/SP**	0,05	0,07	0,23	0,65
**20 phm CCB/SP**	0,07	0,07	0,23	0,63
**25 phm CCB/SP**	0,08	0,07	0,22	0,62

[a] $\rho_{CCB} = 1.8$ g/cm^3
[b] $\rho_{CMC} = 1.6$ g/cm^3
[c] $\rho_{glycerol} = 1.2$ g/cm^3
[d] $\rho_{BSF\ protein} = 0.9$ g/cm^3 (from Mshayisa et al. [4])
* Determined considering complete dehydration of the films.
** For composites containing CCB/SP, the CCB content was taken as the 89% of adduct weight. SP volumetric fraction was considered negligible.

Text S.8. Experimental procedure for swelling tests and determination of water interaction parameters.

Films were weighed at time zero (W_0), then submitted to dehydration in a ventilated oven at 80°C for 24h and weighed again (W_D). Films were next immersed in Milli-Q water enriched with benzalkonium chloride (BAC) (100 µg/mL final concentration) to prevent microbial or fungi growth after several days of immersion. Weights of swollen films (gently dabbed with absorbing paper) were taken after 6 h soaking (W_S). Films were eventually dried in a ventilated oven at 80°C for 24h and weighed to conclude the test (W_F). The test was conducted at least in triplicate for each tested sample. The following film-water interaction parameters were calculated as follows.

Moisture content (M.C.%), i.e., the amount of water naturally present in films after the preparation process (Equation 3):

$$M.C.\% = \frac{W_0 - W_D}{W_0} \quad (3)$$

Water uptake (W.U.%), i.e. the amount of water absorbed by the film after 6h of immersion in water (Equation 4):

$$W.U.\% = \frac{W_S - W_G}{W_G} \quad (4)$$

Film solubility (F.S.%), i.e. the amount of material lost (i.e. solubilized or precipitated from the sample) after the swelling test (Equation 5):

$$F.S.\% = \frac{W_D - W_F}{W_D} \quad (5)$$

Volumetric variation ($\Delta V\%$), i.e. the variation of volume of the film after 6h of immersion in water compared to the initial volume of the dry film (Equation 6):

$$\Delta V\% = \frac{V_S - V_D}{V_D} \quad (6)$$

Where V_S is the volume of the swollen sample and V_D is the volume of the dry sample.

W_G is the weight of the film without glycerol (i.e., W_D subtracted of the glycerol amount, known for each formulation). This correction was due considering that glycerol is completely removed from the film after 6h, as shown in recent publication by some of the authors [3].

Sol fraction, Gel fraction and Sol/Gel fraction were calculated as follows (Equations 7,8,9):

$$Sol\, fraction\ (\%) = \frac{W_D - W_F}{W_D} \quad (7)$$

$$Gel\, fraction\ (\%) = \frac{W_F}{W_D} \quad (8)$$

$$Sol/Gel\, fraction = \frac{Sol\, fraction}{Gel\, fraction} \quad (9)$$

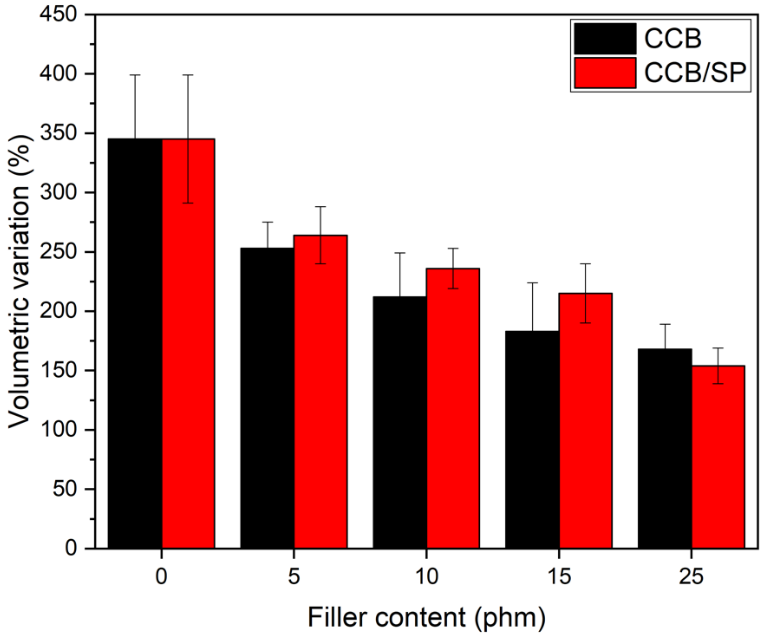

Figure S.7. Volumetric variation (ΔV%) as a function of the filler content for both set of bio-nanocomposite films (black columns for CCB and red columns for CCB/SP).

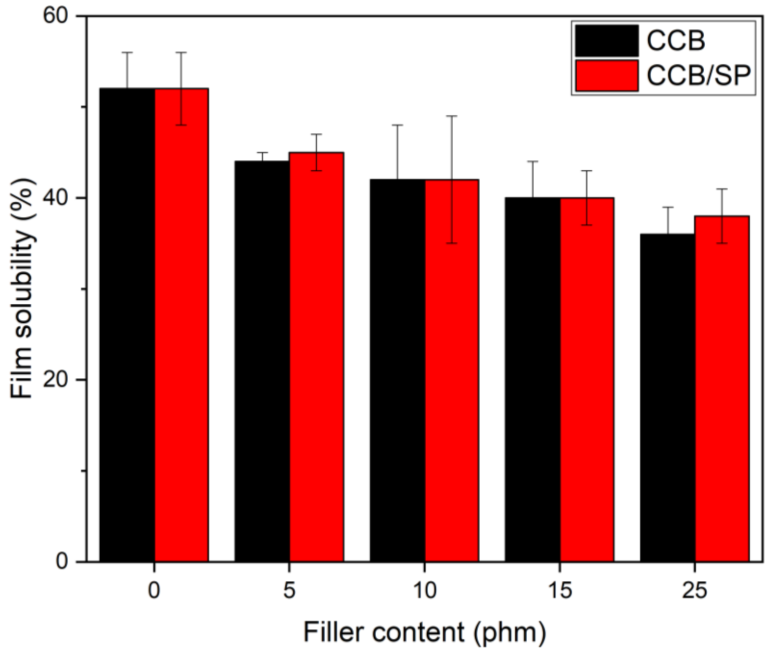

Figure S.8. Film solubility (FS%) as a function of the filler content for both sets of bio-nanocomposite films (black columns for CCB and red columns for CCB/SP).

Table S.5. Sol/Gel fraction data for the two tested sets of bio-nanocomposites as a function of their filler contents.

	Filler content [php]				
	**0**	**5**	**10**	**15**	**25**
**CCB**	1.08	0.79	0.72	0.66	0.57
**CCB/SP**	1.08	0.82	0.74	0.66	0.64

Text S.9. Determination of the Kraus constant from the Kraus model.

The Kraus plot analysis has been often used to assess the polymer-filler interaction in elastomeric composites.[5–7] Indeed, according to the binary system shown below ((Equation 10 and 11), it is possible to quantitatively evaluate the extent of the filler-matrix interaction in a composite material when this is exposed to a solvent promoting its swelling. In particular, the model elaborated by Kraus proposes that stronger filler-matrix interactions are associated with higher Kraus constants (C).

$$\begin{cases} \frac{v_{r0}}{v_{rf}} = 1 - m\left(\frac{\phi_f}{1-\phi_f}\right) \\ C = \frac{|m| - v_{r0} + 1}{3\left(1 - v_{r0}^{1/3}\right)} \end{cases} \quad (10)\ (11)$$

where v_{r0} is the volume fraction of matrix in the swollen film without any filler, v_{rf} is the volume fraction of matrix in the swollen film with filler, ϕ_f is the volume fraction of the filler and *m* is the experimental slope of the linear regression between $\frac{v_{r0}}{v_{rf}}$ and $\frac{\phi_f}{1-\phi_f}$.

v_{r0} and v_{rf} were calculated as follows:

$$v_{rf} = \frac{W_m}{W_m + \frac{\rho_M}{\rho_A}W_A + \frac{\rho_M}{\rho_F}W_F} \quad (12)$$

Where W_m is the weight of the matrix (BSF proteins + CMC), W_A is the weight of absorbed water, W_f is the weight of the filler, ρ_M is the density of the film matrix (i.e., 1.017 g/cm^3 (weighted average between BSF proteins (0.9 g/cm^3) and CMC (1.6 g/cm^3) (BSF protein density was from Mshayisa et al .[4]), ρ_A is the density of water (i.e., 1 g/cm^3), and ρ_f is the density of the filler (i.e., 1.8 g/cm^3, from material data sheet). v_{r0} can be obtained setting $W_f = 0$.

Values of v_{r0}/v_{rf} was plotted with respect to $\phi_f/(1-\phi_f)$ values, and the best line through the points was found by linear regression. The slope of the linear regression (*m*) was implemented in the Equation 11 to determine the Kraus constant (*C*).

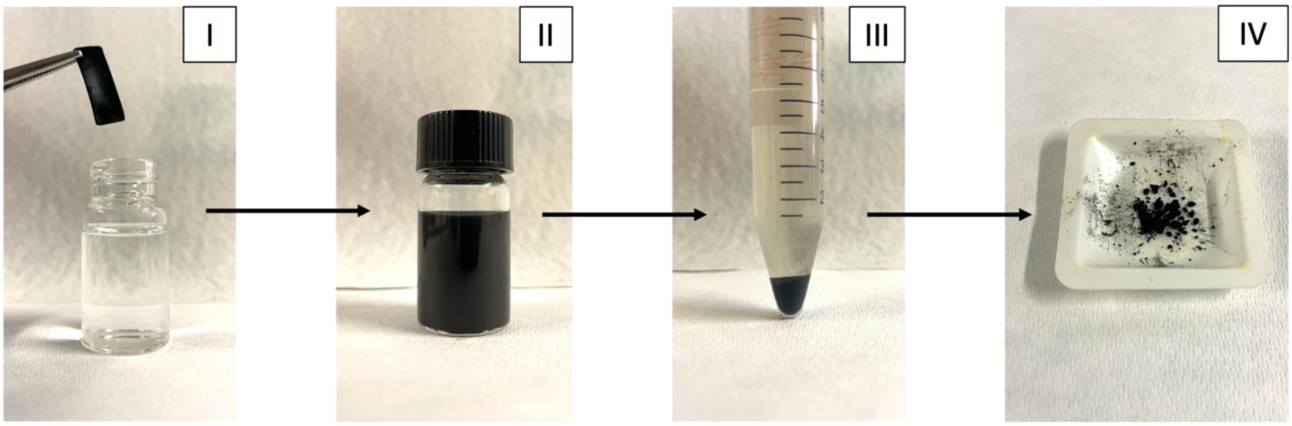

Figure S.9. Degradation of the BSF protein nanocomposite and recovery of CCB. (I) starting film sample; (II) the film is degraded in 1M HCl or 1% pepsin-water solutions; (III) CCB is unstable in water solutions and rapidly precipitate after a centrifugation step; (IV) CCB was recovered by resuspension in acetone and subsequent evaporation of the solvent.

Table S.6 Collection of current nanocomposite materials and their performances as components for flexible electronics applications.

**Material**	**Conductivity, σ [S/cm]**	**Filler content [phm[a]]**	**Proposed application**	**Ref.**
Bio-based polymers				
BSF proteins, functionalized CB	0.9×10^{-2}	25	Motion sensing, electrostatic discharge packaging	This work
Keratin, graphene	10 *	30	Resistor, capacitor, inductor, conductive ink	2
Soy protein, starch nanocrystals, cRGO	6.58×10^{-7}	1	Humidity sensor, conductive element, supercapacitor	8
β-lactoglobulin, cRGO	5.1×10^{-1}	12.5	Humidity sensor, enzyme activity biosensor	9
Starch/polyester, graphene	10 *	50	Conductive ink	10
Starch, graphene	9.7×10^{-4}	1.8	Not specified	11
Starch, graphene	3.9×10^{-4}	2	Not specified	12
Starch, SWCNT	10^{-3}	n.a.	Not specified	13
Oil-based polymers				
Nylon, CB	1.3×10^{-2}	22	Antistatic fibres for fabrics	14
PET, CB	9.5×10^{-2}	18	Antistatic fibres for fabrics	14
PET, exfoliated graphite	10^{-6}	7.5	Not specified	15
PET, graphite	1.6×10^{-5}	17	Not specified	16
Poly(1,4-cis-isoprene), CB+CNT	10^{-4}	15	Not specified	17
PP, CB	4.1×10^{-2}	22	Antistatic fibres for fabrics	14
PP, CB	1	n.a.	Not specified	18
PP, functionalized CB	1.19×10^{-4}	20	Not specified	19

[a] phm: parts per hundred matrix
[b] cRGO: chemically reduced graphene oxide
[c] n.a.: data not available from text
[*] ohm/sq

Text S.10. Experimental setup for the measurement of R_V.

Measurements of R_v, taken on at least 4 defined film regions for each tested sample, were collected with a four-point probe set, using two out of four gold-coated probe clips to clamp each film between the top and the bottom side (**Figure a**).

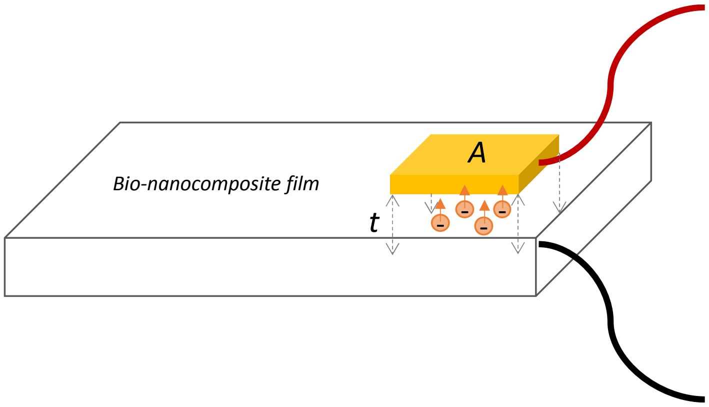

Figure a. Experimental setup for the measurement of volumetric resistance (R_v) in bio-nanocomposite films.

The volumetric resistivity (ρ) was calculated applying the second Ohm law equation (14):

$$\rho = \frac{R \times A}{t} \quad (14)$$

Where A is the probe/material contact area (A = 37.1 mm^2) and t is the film thickness as an average of four distinct measurements taken on different regions of each sample. Electrical conductivity (σ) was obtained as the inverse of the obtained electrical resistivity.

Text S.11. Percolation theory model applied to presented materials.

The electrical conductivity of prepared bionanocomposites followed with good approximation the percolation theory model [10,20,21], whose equation is here reported:

$$\sigma = \sigma_0(\phi - \phi_c)^\tau \qquad \text{valid for} \quad \phi > \phi_c$$

The equation was linearized in the *log* space, as follows:

$$log\ (\sigma) = log\ (\sigma_0) + \tau \cdot log(\phi - \phi_c) \qquad \text{valid for} \quad \phi_f > \phi_c$$

where σ is the electrical conductivity of the composite, σ_0 is a proportionality constant characteristic of the material, ϕ is the filler volumetric fraction, ϕ_c is the percolation threshold, and τ is the universal exponent, with typical values ranging from 1.6 to 2.0 in three-dimensional systems [10,20]. Briefly, the electrical percolation theory aims to explain how electrical conductivity emerges in a composite material when a sufficient amount of a conductive component is added to an insulating matrix. The key concept is the percolation threshold (ϕ_c), which refers to the minimum amount of the conductive component necessary to substantially enhance the composite's electrical conductivity compared to the non-conductive phase. In principle, this model is known to well suit composite materials with spherical filler particles [20,21]. In our case, whilst CCB is nanometrically characterized by turbostratic graphene layers which aggregate to form nano-sized spheres, the micro-structure of CCB is usually branched, following aggregation of the nanospheres. Here, the model was applied on both sets of bionanocomposites seeking for τ values characteristic of 3D networks (i.e., τ = 2) [10,20]. Electrical percolation thresholds of $\Phi_C = 0.061$ and $\Phi_C = 0.043$ were then determined for CCB and CCB/SP containing bionanocomposites, respectively, being in line with graphical data (see Figure 6B).

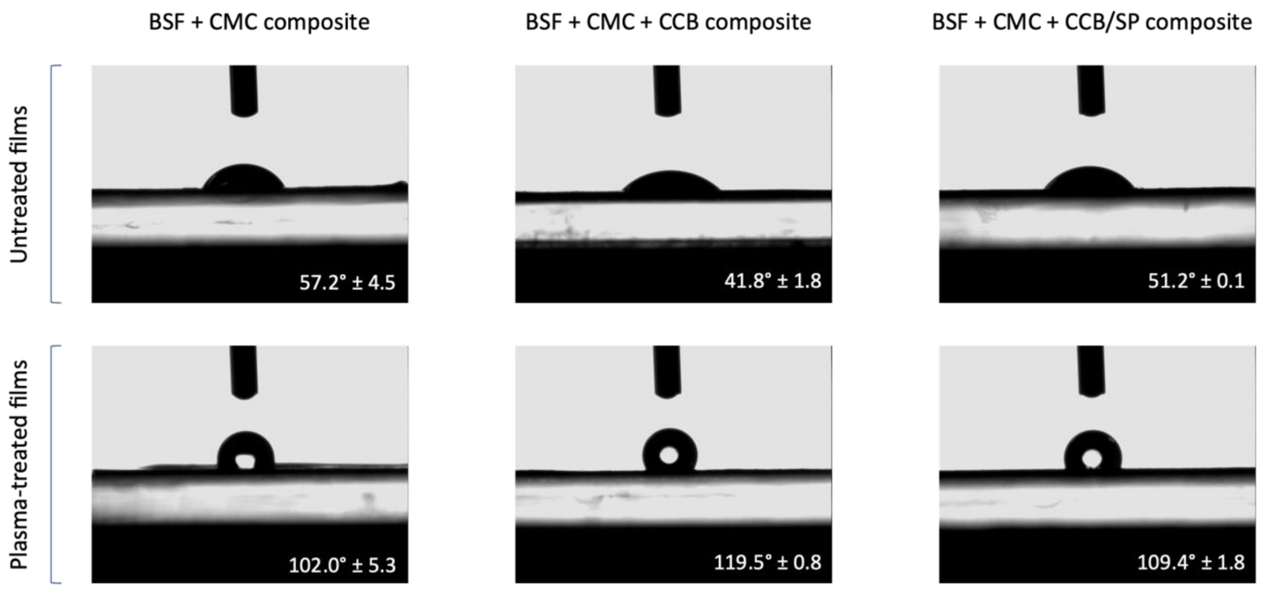

Figure S.10. Static water contact angle (WCA) pictures and measurements for 25 phm CCB and CCB/SP-containing nanocomposites.

References

(1) Pennetta, C.; Floresta, G.; Graziano, A. C. E.; Cardile, V.; Rubino, L.; Galimberti, M.; Rescifina, A.; Barbera, V. Functionalization of Single and Multi-Walled Carbon Nanotubes with Polypropylene Glycol Decorated Pyrrole for the Development of Doxorubicin Nano-Conveyors for Cancer Drug Delivery. *Nanomaterials* **2020**, *10* (6), 1073. https://doi.org/10.3390/NANO10061073.

(2) Cataldi, P.; Condurache, O.; Spirito, D.; Krahne, R.; Bayer, I. S.; Athanassiou, A.; Perotto, G. Keratin-Graphene Nanocomposite: Transformation of Waste Wool in Electronic Devices. *ACS Sustain Chem Eng* **2019**, *7* (14), 12544–12551. https://doi.org/10.1021/acssuschemeng.9b02415.

(3) Bruno, D.; Orlando, M.; Testa, E.; Carnevale Miino, M.; Pesaro, G.; Miceli, M.; Pollegioni, L.; Barbera, V.; Fasoli, E.; Draghi, L.; Baltrocchi, A. P. D.; Ferronato, N.; Seri, R.; Maggi, E.; Caccia, S.; Casartelli, M.; Molla, G.; Galimberti, M. S.; Torretta, V.; Vezzulli, A.; Tettamanti, G. Valorization of Organic Waste through Black Soldier Fly: On the Way of a Real Circular Bioeconomy Process. *Waste Management* **2025**, *191*, 123–134. https://doi.org/10.1016/J.WASMAN.2024.10.030.

(4) Mshayisa, V. V.; Van Wyk, J.; Zozo, B. Nutritional, Techno-Functional and Structural Properties of Black Soldier Fly (Hermetia Illucens) Larvae Flours and Protein Concentrates. *Foods* **2022**, *11* (5), 724. https://doi.org/10.3390/FOODS11050724.

(5) Yang, B.; Nagarajan, B.; Mertiny, P. Characterization of Swelling Behavior of Carbon Nano-Filler Modified Polydimethylsiloxane Composites. *Journal of Elastomers and Plastics* **2021**, *53* (8), 955–974. https://doi.org/10.1177/00952443211006156.

(6) Kim, D. Y.; Park, J. W.; Lee, D. Y.; Seo, K. H. Correlation between the Crosslink Characteristics and Mechanical Properties of Natural Rubber Compound via Accelerators and Reinforcement. *Polymers (Basel)* **2020**, *12* (9), 1–14. https://doi.org/10.3390/polym12092020.

(7) Mohapatra, S.; Alex, R.; Nando, G. B. Cardanol Grafted Natural Rubber: A Green Substitute to Natural Rubber for Enhancing Silica Filler Dispersion. *J Appl Polym Sci* **2016**, *133* (8), 1–16. https://doi.org/10.1002/APP.43057.

(8) Zhu, G.; Dufresne, A.; Lin, N. Humidity-Sensitive and Conductive Nanopapers from Plant-Derived Proteins with a Synergistic Effect of Platelet-Like Starch Nanocrystals and Sheet-Like Graphene. *ACS Sustain Chem Eng* **2017**, *5* (10), 9431–9440. https://doi.org/10.1021/acssuschemeng.7b0257.

(9) Li, C.; Adamcik, J.; Mezzenga, R. Biodegradable Nanocomposites of Amyloid Fibrils and Graphene with Shape-Memory and Enzyme-Sensing Properties. *Nat Nanotechnol* **2012**, *7* (7), 421–427. https://doi.org/10.1038/nnano.2012.62.

(10) Cataldi, P.; Bayer, I. S.; Bonaccorso, F.; Pellegrini, V.; Athanassiou, A.; Cingolani, R. Foldable Conductive Cellulose Fiber Networks Modified by Graphene Nanoplatelet-Bio-Based Composites. *Adv Electron Mater* **2015**, *1* (12), 1–8. https://doi.org/10.1002/aelm.201500224.

(11) Zheng, P.; Ma, T.; Ma, X. Fabrication and Properties of Starch-Grafted Graphene Nanosheet/ Plasticized-Starch Composites. *Ind Eng Chem Res* **2013**, *52* (39), 14201–14207. https://doi.org/10.1021/ie402220d.

(12) Gürler, N.; Torğut, G. Graphene-Reinforced Potato Starch Composite Films: Improvement of Mechanical, Barrier and Electrical Properties. *Polym Compos* **2021**, *42* (1), 173–180. https://doi.org/10.1002/PC.25816.

(13) Prusty, G.; Das, R.; Swain, S. K. Influence of Functionalized Single-Walled Carbon Nanotubes on Morphology, Conducting and Oxygen Barrier Properties of Poly (Acrylonitrile-Co-Starch). *Compos B Eng* **2014**, *62*, 236–241. https://doi.org/10.1016/J.COMPOSITESB.2014.03.006.

(14) Choi, H. J.; Kim, M. S.; Ahn, D.; Yeo, S. Y.; Lee, S. Electrical Percolation Threshold of Carbon Black in a Polymer Matrix and Its Application to Antistatic Fibre. *Sci Rep* **2019**, *9* (1), 1–12. https://doi.org/10.1038/s41598-019-42495-1.

(15) Li, M.; Jeong, Y. G. Poly(Ethylene Terephthalate)/Exfoliated Graphite Nanocomposites with Improved Thermal Stability, Mechanical and Electrical Properties. *Compos Part A Appl Sci Manuf* **2011**, *42* (5), 560–566. https://doi.org/10.1016/j.compositesa.2011.01.015.

(16) Alshammari, B. A.; Al-Mubaddel, F. S.; Karim, M. R.; Hossain, M.; Al-Mutairi, A. S.; Wilkinson, A. N. Addition of Graphite Filler to Enhance Electrical, Morphological, Thermal, and Mechanical Properties in Poly (Ethylene Terephthalate): Experimental Characterization and Material Modeling. *Polymers (Basel)* **2019**, *11* (9). https://doi.org/10.3390/polym11091411.

(17) Galimberti, M.; Coombs, M.; Riccio, P.; Riccò, T.; Passera, S.; Pandini, S.; Conzatti, L.; Ravasio, A.; Tritto, I. The Role of CNTs in Promoting Hybrid Filler Networking and Synergism with Carbon Black in the Mechanical Behavior of Filled Polyisoprene. *Macromol Mater Eng* **2013**, *298* (2), 241–251. https://doi.org/10.1002/mame.201200075.

(18) Gong, T.; Peng, S. P.; Bao, R. Y.; Yang, W.; Xie, B. H.; Yang, M. B. Low Percolation Threshold and Balanced Electrical and Mechanical Performances in Polypropylene/Carbon Black Composites with a Continuous Segregated Structure. *Compos B Eng* **2016**, *99*, 348–357. https://doi.org/10.1016/j.compositesb.2016.06.031.

(19) Shepherd, C.; Hadzifejzovic, E.; Shkal, F.; Jurkschat, K.; Moghal, J.; Parker, E. M.; Sawangphruk, M.; Slocombe, D. R.; Foord, J. S.; Moloney, M. G. New Routes to Functionalize Carbon Black for Polypropylene Nanocomposites. *Langmuir* **2016**, *32* (31), 7917–7928. https://doi.org/10.1021/acs.langmuir.6b02013.

(20) Zare, Y.; Rhee, K. Y.; Park, S. J. Advancement of the Power-Law Model and Its Percolation Exponent for the Electrical Conductivity of a Graphene-Containing System as a Component in the Biosensing of Breast Cancer. *Polymers (Basel)* **2022**, *14* (15). https://doi.org/10.3390/polym14153057.

(21) Zhang, H.; Dou, C.; Pal, L.; Hubbe, M. A. Review of Electrically Conductive Composites and Films Containing Cellulosic Fibers or Nanocellulose. *Bioresources* **2019**, *14* (3), 7494–7542. https://doi.org/10.15376/biores.14.3.7494-7542.
